# Exploring the Influence of Date Palm Cultivars on Soil Microbiota

**DOI:** 10.1007/s00248-024-02415-x

**Published:** 2024-08-01

**Authors:** Pedro Ferreira, Mohamed Ali Benabderrahim, Hammadi Hamza, Alexis Marchesini, Mokhtar Rejili, Joana Castro, Rui M. Tavares, Daniela Costa, Federico Sebastiani, Teresa Lino-Neto

**Affiliations:** 1 Centre of Molecular and Environmental Biology (CBMA), Department of Biology, University of Minho, Campus of Gualtar, 4710-057 Braga, Portugal; 2 Faculty of Sciences of Tunis, Department of Biology, University of Tunis EL Manar, 2092 Tunis, Tunisia; 3 Drylands and Oases Cropping Laboratory. Arid Areas Institute (IRA), 4119 Medenine, Tunisia; 4 Institute for Sustainable Plant Protection, National Research Council, Via Madonna del Piano 10, 50019 Sesto Fiorentino, FI Italy; 5 Research Institute On Terrestrial Ecosystems, National Research Council, Porano, Italy; 6 National Biodiversity Future Center, Palermo, Italy; 7 Department of Life Sciences, Al Imam Mohamed Ibn Saud Islamic University (IMSIU), 11623 Riyadh, Saudi Arabia

**Keywords:** *Phoenix dactylifera* L., Cultivars, Soil microbiome, Plant tolerance, Sustainability

## Abstract

Plants thrive in diverse environments, where root-microbe interactions play a pivotal role. Date palm (*Phoenix dactylifera* L.), with its genetic diversity and resilience, is an ideal model for studying microbial adaptation to different genotypes and stresses. This study aimed to analyze the bacterial and fungal communities associated with traditional date palm cultivars and the widely cultivated “Deglet Nour” were explored using metabarcoding approaches. The microbial diversity analysis identified a rich community with 13,189 bacterial and 6442 fungal Amplicon Sequence Variants (ASVs). Actinobacteriota, Proteobacteria, and Bacteroidota dominated bacterial communities, while Ascomycota dominated fungal communities. Analysis of the microbial community revealed the emergence of two distinct clusters correlating with specific date palm cultivars, but fungal communities showed higher sensitivity to date palm genotype variations compared to bacterial communities. The commercial cultivar “Deglet Nour” exhibited a unique microbial composition enriched in pathogenic fungal taxa, which was correlated with its genetic distance. Overall, our study contributes to understanding the complex interactions between date palm genotypes and soil microbiota, highlighting the genotype role in microbial community structure, particularly among fungi. These findings suggest correlations between date palm genotype, stress tolerance, and microbial assembly, with implications for plant health and resilience. Further research is needed to elucidate genotype-specific microbial interactions and their role in enhancing plant resilience to environmental stresses.

**Supplementary Information:**

The online version contains supplementary material available at 10.1007/s00248-024-02415-x.

## Background

As sessile organisms, plants have evolved mechanisms and strategies to successfully overcome the adversities present in a constantly changing environment [1]. One of these strategies relies on the interaction of plant roots with soil microbial communities [2]. The soil under root influence harbors a vast number of microorganisms, which can promote plant growth, nutrient acquisition, and stress alleviation [3, 4]. The structure of the rhizospheric microbiome is influenced by different factors, including the soil type, plant species, developmental stage, and genotype [5]. Indeed, microbial communities are shaped by plant exudates secreted into the rhizosphere, which are also able to reinforce beneficial interactions [6]. In fact, the rhizospheric microbiome is so important for the plant that about 5–21% of total photosynthetically locked carbon is allocated to the soil through exudates [7]. In exchange, soil microorganisms are able to interact with plants, providing them access to nutrients that otherwise would not be available, stimulating plant growth, and increasing resistance to phytopathogens [8, 9]. Due to their importance for plant health and adaptation capacity, learning more about rhizospheric communities could be the basis of new sustainable agricultural practices. Therefore, greater effort should be given to research plants that are already adapted to extreme environments to understand their microbial community structure and how they thrive in harsh conditions. The elucidation of the microbial community could potentially find sustainable solutions to cope with the new environmental conditions resulting from climate change.

Date palm (*Phoenix dactylifera* L.) is recognized as one of the oldest fruit crops and is considered the most important fruit crop in the arid regions of the Arabian Peninsula, North Africa, and Middle East [10]. This crop is renowned for its extensive genetic diversity, encompassing around 5000 cultivars, and this genetic pool remains largely unexplored and poorly understood [11]. Seedling propagation results in a heterogeneous population, which often leads to low yields and poor-quality fruits [12]. To overcome such disadvantages and meet the increased demand for fruits, *P. dactylifera* is commonly propagated by offshoots, with in vitro tissue culture also being a common solution [13]. As a consequence, certain cultivars have gained prominence due to the widespread acceptance of their fruits in different worldwide markets, such as “Deglet Nour,” “Medjool,” and “Barhee” [14]. The cultivation of these popular cultivars in monoculture within the oasis has led to the genetic erosion of date palm [15]. Concerns are also arising regarding soil erosion and its impact on date palm oases, particularly in the context of climate change adaptation [16]. Date palms form symbiotic relationships with soil microbes [17] and are known to survive under harsh conditions [18]. They are cultivated in arid regions from the Mediterranean Basin, where optimal daily temperatures for date palms range from 28 to 41 °C. Remarkably, they can withstand temperatures as high as 50 °C and tolerate brief periods of temperatures as low as − 5 °C [19, 20]. These unique characteristics make *P. dactylifera* an excellent model for studying the role of the rhizospheric microbiome in mitigating extreme abiotic stresses. Advancements in high-throughput technologies are enhancing efforts to understand the soil microbial communities associated with date palms [e.g., [21, 22]]. Metabarcoding studies targeted to bacteria and fungi are providing insights into how genotypes, environmental conditions and cultural practices shape microbial communities [22–25]. While some studies report that date palm cultivars significantly influence the rhizosphere soil by affecting microbial communities [25] others describe varying effects of different genotypes on bacterial and fungal communities [24]. Investigating several traditional date palm cultivars, together with largely marketed cultivars, could give new hints into the impact of genotypes on microbial community shaping. With this study, we aim to analyze the bacterial and fungal communities present in the soils influenced by date palm and investigate the effects of date palm genetic diversity on microbial community composition. To achieve these objectives, we used an experimental plot where distinct date palm genotypes were cultivated side-by-side, thus minimizing the influence of edaphoclimatic variations. By examining a diverse range of traditional date palm cultivars and a widely marketed cultivar, we anticipate that our study will provide new insights into how genotype impacts microbial community structure.

## Materials and Methods

### Site Description and Sampling

Soils were sampled from date palm trees (*Phoenix dactylifera* L*.*) at a single oasis situated in the Tozeur region, located in Southwest of Tunisia (33.954855N, 8.026501E). This region displays a subtropical desert climate with mild winter and hot summer, annual precipitation around 96 mm/year, and an average annual temperature of 22.0 °C. This study was conducted in an experimental plot (100 m × 90 m) established over 30 years ago as a conservation zone dedicated to preserving the genetic diversity of date palms. The plot accommodated nine different cultivars (“Bejou,” “Besser Helou,” “Deglet Nour,” “Gondi,” “Gosbi,” “Kenta,” “Lagou,” “Tezerzayet Kahla,” and “Tezerzayet Safra”), all growing side by side (SI Fig. 1) and subjected to identical edaphoclimatic conditions. The soils in the area are poorly developed, with a sandy loam texture and an average pH of 7.4. Moreover, no agricultural treatments were applied, ensuring the preservation of the natural variability in soil chemical properties*.* Irrigation was performed with water containing 50 mM of salt. All date palms were of similar age and were separated from each other at least 10 m. Four independent soil samples were collected from each cultivar in the influential zone of date palm roots. Each sample corresponded to a single tree and resulted from the combination of three subsamples taken from identical distances from a date palm trunk (approximately 1 m). Soil sampling involved removing the superficial layer (approximately 20 cm) and collecting soil from the 10 to 20-cm-depth horizon to capture the soil under the influence of superficial roots. All soil samples were stored at − 20 °C.

### DNA Extraction and Sequencing

Before DNA extraction, soils were sieved twice, first with a 5-mesh (4-mm^2^ sieve opening) and then with a 10-mesh size (2 mm^2^ sieve opening). DNA extraction was performed for each combined soil sample using the *DNeasy PowerSoil Pro* kit (Qiagen, Germany), according to the manufacturer recommendations. Amplification capacity was assessed by a PCR assay with the following primers: *799F* (AACMGGATTAGATACCCKG [26]) and *1492R* (GGTTACCTTGTTACGACTT [26]) for the bacterial *16S V5-V9* region; *ITS1-F* (CTTGGTCATTTAGAGGAAGTAA [27]) and *ITS2* (GCTGCGTTCTTCATCGATGC [27]) for fungal internal transcribed spacer 1 (*ITS1*) region. The PCR reaction mixtures (25 μl) contained 1 × *Complete NH*_*4*_ reaction buffer (Bioron GmbH, Germany), 200 μM of each dNTP (NZYTech, Portugal), 5 μM of each primer, 1 μl of DNA template (20 ng/μl), and 1.25 U *DFS-Taq DNA Polymerase* (Bioron GmbH, Germany). Amplifications were performed in a *T100™ Thermal Cycler* (Bio-Rad, USA), using the following protocol: initial denaturation for 4 min at 94 °C; 35 cycles of denaturation during 30 s at 94 °C, annealing during 30 s at 54 °C (*16S*) or 56 °C (*ITS*) and extension during 30 s at 72 °C; final elongation at 72 °C for 10 min. PCR products were run on a 1% (w/v) agarose gel stained with *Green Safe Premium* (NZYTech, Portugal). DNA samples that resulted in amplification were quantified using a fluorescent DNA quantification assay with *dsDNA HS Assay* kit (Thermo Fisher Scientific, USA) and detected with a *Qubit 3.0 Fluorometer* (Thermo Fisher Scientific, USA). DNA samples presenting a concentration higher than 20 ng/µl were sequenced using a service provider (GenoInSeq, Portugal). Sequencing was performed using an *Illumina MiSeq®* sequencer with V3 chemistry through paired-end sequencing (2 × 250 bp) and a sequence depth of 150,000 reads per sample. For bacterial communities, the used forward primer was *799F* (5′-AACMGGATTAGATACCCKG) and the reverse primer *1193R* (5′-ACGTCATCCCCACCTTCC) [26, 28]. Regarding fungal communities the reverse primer *ITS3NGS1_F* (5′-CATCGATGAAGAACGCAG) and a pool of forward primers (*ITS3NGS2_F*: 5′-CAACGATGAAGAACGCAG; *ITS3NGS3_F*: 5′-CACCGATGAAGAACGCAG, *ITS3NGS4_F*: 5′-CATCGATGAAGAACGTAG, *ITS3NGS5_F*: 5′-CATCGATGAAGAACGTGG-3′; ITS3NGS10_F: 5′-CATCGATGAAGAACGCTG) were used [29].

### Processing of Sequencing Data

Raw reads were obtained from *Illumina MiSeq®* in fastq format. Sequencing reads generated in this study have been submitted to the NCBI Sequence Read Archive with the accession number PRJNA1109279. Reads with less than 100 bases were discarded with *PRINSEQ* (version 0.20.4). Trimming based on quality scores was performed using default parameters in *Sickle*, version 1.33 [30]. Correction of errors in reads was performed using Bayeshammer module from *SPAdes* package version 3.15.5 [31]. The merging of overlapping paired-end reads and filtering was achieved using *Usearch* version 11.0.67 [32]. *Fastq-mcf* from *ea-utils* package (version 1.04.676 [33]) was used to filter merged reads based on sequence length. The software *micca* version 1.7.2 [34] was used to create a single fastq file with the package *micca merge*. To discard sequences with an expected error rate greater than 1%, the *micca filter* package was used. Additionally, *mica otu* allowed clustering amplicon sequences variants (ASVs), by using UNOISE3 protocol and chimeric sequences removal. *Micca classify* package was used to assign taxonomy to each ASV: qiime2-compatible SILVA138.99 [35] for bacteria and UNITE database version 9.0 [36] for fungi. Sequences of unclassified ASVs were further searched using NCBI-BLAST, so their taxonomic classification could be understood. Those ASVs assigned as unclassified were removed from the bacterial dataset. For fungal datasets, ASV assigned as unclassified, Viridiplantae, or others not corresponding to a fungal taxonomy were removed.

### Date Palm Genetic Diversity Analysis

Leaf samples were collected from each of the nine cultivar trees, with four replicates per cultivar, totaling 36 sample trees. Total DNA was extracted DNA extraction was performed for each combined soil sample using the *Quick-DNA fungal/bacterial* kit (Zymo research, USA). A whole genome sequencing (WGS) approach in paired-end mode (2 × 150 bp reads) on an *Illumina HiSeq 4000* platform was used to identify single nucleotide polymorphisms (SNPs). Library construction and sequencing were performed by Novogene Co., Ltd. (Beijing, China). Sequencing reads produced during this study have been deposited at the NCBI Short Read Archive under the accession number PRJNA1134368. Quality control of the reads was assessed with *FASTQC version 0.11.8* [37]. Reads were then processed with *fastp version 0.20.0* [38] for trimming and adapters removal; read tails with a mean Phred-quality score < 15 over a 4-bp sliding window were trimmed. Subsequently, the clean reads were mapped to the date palm reference genome (GenBank accession number: NC_052392.1) using the *Burrows-Wheeler Aligner*, *BWA-MEM*, *v0.7.17* [39] with “-R” flag for RG tag implementation. Alignments in sam format were sorted, marked for duplicates, indexed and compressed in bam format using *SAMTOOLS version 1.9* [40]; only mapped reads were used for downstream analysis and BAM files were validated using the ValidateSamFile of *PICARD v2.25.4* (http://broadinstitute.github.io/picard). The observed average depth of coverage was computed for each sample using the depth command of *SAMTOOLS*, we excluded samples with average depth of coverage < 3.0. Variant calling was performed using the *Haplotype Calle*r (in -ERC GVCF mode) and *GenotypeGVCFs* tools *in GATK v4.2.3.0* [41]. We applied a rigorous, multistep variant filtering procedure, which can be summarized as follows: (1) Only SNPs and high quality sequencing (we filtered SNPs using *GATK VariantFiltration* tool, by excluding variants which were not a SNP (i.e. indels), and by excluding SNPs matching at least one of the following criteria: significant Fisher strand test (FS > 60); Variant Confidence/Quality by Depth (QD) < 2; root mean square of the Mapping Quality (MQ) < 40; root mean square of the mapping quality (MQRankSum) <  − 12.5 or significant read position bias (ReadPosRankSum <  − 8.0); strand bias (StrandOddsRatio; SOR) > 3.0); (2) only biallelic SNPs were retained, using vcftools, (“–min-alleles 2” and “–max-alleles 2” flags), and SNPs with depth of coverage (DP) ≤ 4 × or ≥ 12 × were excluded (“–min-meanDP 4” and “–max-meanDP 12” flags); (3) we removed SNPs with > 95% missing data using vcftools (option –max-missing 0.95) and kept only variants with a minor allele frequency of at least 0.05 using vcftools (option –maf 0.05); (4) we removed individuals with > 10% of missing data using vcftools. Distance matrices between tree samples were calculated using PLINK 1.9 [42] via its identity-by-state (IBS) and Hamming distance algorithms. VCF2PCACluster [43] was used for PCA-based clustering with the Normalized_IBS option.

### Microbial Diversity and Similarity Analysis

The following analyses were performed in *RStudio* version 4.2.2, using the package *microeco* [44], except when stated otherwise. Bacterial and fungal datasets were subsampled using the function *rarefy_samples()*, considering the lowest number of sequences found in all samples (14,549 sequences for bacterial dataset, found in a *Kenta* cultivar sample—*KT1*; 22,688 sequences for fungal dataset, found in *Deglet Nour* cultivar sample—*DEN4*; Tables S1 and S1). Rarefied ASV datasets were used for downstream analyses. Rarefaction curves were calculated to determine the sampling effort by using *trans_rarefy()* function. Bacterial and fungal ASVs abundance and richness were determined for different cultivars using *trans_abund()* function. Richness (*S*) and diversity parameters [Gini-Simpson’s index (1-*D*) and Shannon’s index (*H*′)] in the different datasets were plotted using *cal_alphadiv()* function. These ecological parameters were compared between samples from different date palm cultivars by ANOVA, performed with *cal_diff()* function.

Non-metric multidimensional scaling (NMDS) was performed with Bray–Curtis dissimilarity coefficient using *trans_beta()* function. The ordination graph was obtained through the *calordination()* function. Kruskal’s stress was used to measure the model’s goodness of fit (stress values between 0.1 and 0.2 represent a good ordination [45]). To evaluate differences between cultivars and genetic clusters, PERMANOVA was performed with the Bray–Curtis coefficient (999 permutations) using *calmanova()* function. Statistical differences in the relative abundance of genera across distinct date palm cultivars were evaluated through ANOVA testing using *transdiff()* function. A Mantel test was performed using the distance matrix of each microbial community and the distance matrix produced by the genetic studies. The function *call_mantel()* was used for this analysis. A co-occurrence network was also analyzed using the function *call_network()*. The correlation method used was SPIEC-EASI [46]. Bacterial functional groups were identified using the *FAPROTAX* database [47], whereas fungal functional groups were identified using the *FUNGuild* database [48]. *Phetamap* package was used for heatmap analysis [49], while *microbiome* package [50] was utilized for core microbiome analysis.

### Date Palm Salinity Tolerance Categorization

The categorization of date palm cultivars regarding their salinity tolerance was derived from the extensive experience of date palm farmers. This information was collected as part of the project “*Enrichment of Nefzaoua Oases by Local Date Palm Cultivars Resistant to Salinity and Drought*” funded by the Global Environmental Facility, United Nations Development Program. The cultivars exhibiting the highest salinity tolerance included “Bejou,” “Besser Helou,” and “Kenta.” Those with moderate tolerance were “Gosbi,” “Gondi,” “Tezerzayet Kahla,” “Tezerzayet Safra,” and “Lagou.” The cultivar identified as the most sensitive to salinity was “Deglet Nour.”

## Results

### Date Palm Genetic Diversity

The dataset for the 36 date palm samples (from the nine date palm cultivars) was derived from a larger dataset that includes 220 accessions (see VCF file: https://doi.org/10.6084/m9.figshare.26213120.v1). By resequencing the 36 date palm samples, a total of 916,300,332 raw reads were obtained, resulting in approximately 135 Gb of clean data after filtering out low-quality bases. A total of 864,460,855 reads were accurately reference-mapped against the *P. dactylifera* reference genome, with an average depth of coverage of about 4 × for the 36 samples. After VCF filtering, a total of 1,306,819 biallelic SNPs were retained across the 36 individuals. Based on all polymorphic SNPs generated from the panel, a genetic distance matrix was built among all pairs of accessions, ranging from 0.053 to 0.224, from most closely related to most distant, respectively. As expected, the replicates of the same cultivar showed more homogenous and higher similarity values compared to samples from different cultivars. To further assess the genetic relationships among the selected date palm cultivars, principal component analysis (PCA) was performed. Date palm samples were clustered in 5 groups (SI Fig. 2). The first principal component (PC1), which explained 12.00% of the total variance, clearly separated Cluster 1 (“Tezerzayet Kahla”), Cluster 0 (“Tezerzayet Safra”), and Cluster 3 (“Besser Helou”); the second principal component (PC2), accounted for 11.05% of the total variance, separated Cluster 2 (“Deglet Nour”) and Cluster 4 (“Bejou,” “Gondi,” “Gosbi,” “Lagou,” and “Kenta”).

### Richness and Diversity of Bacterial Communities in Date Palm Soils

A total of 4,682,458 raw reads were obtained from sequencing the *16S* barcode from 36 soil samples, ranging from 67,364 to 220,446 raw reads per sample (SI Table 1). Processed sequences (1,106,836) contained about 0.1% of non-bacterial sequences, which were identified as unclassified ASVs. After removal of these unclassified ASVs, a total of 1,105,841 high-quality bacterial sequences were obtained and clustered into 13,194 ASVs. The dataset was subsampled to reduce the potential bias introduced by different sequencing depths of individual samples. Subsampling was performed based on the sample with the lowest number of classified bacterial sequences (14,549; *KT1* sample) resulting in 13,189 ASVs classified into 25 phyla, 51 classes, 126 orders, 197 families, and 357 genera.

**Fig. 1:**
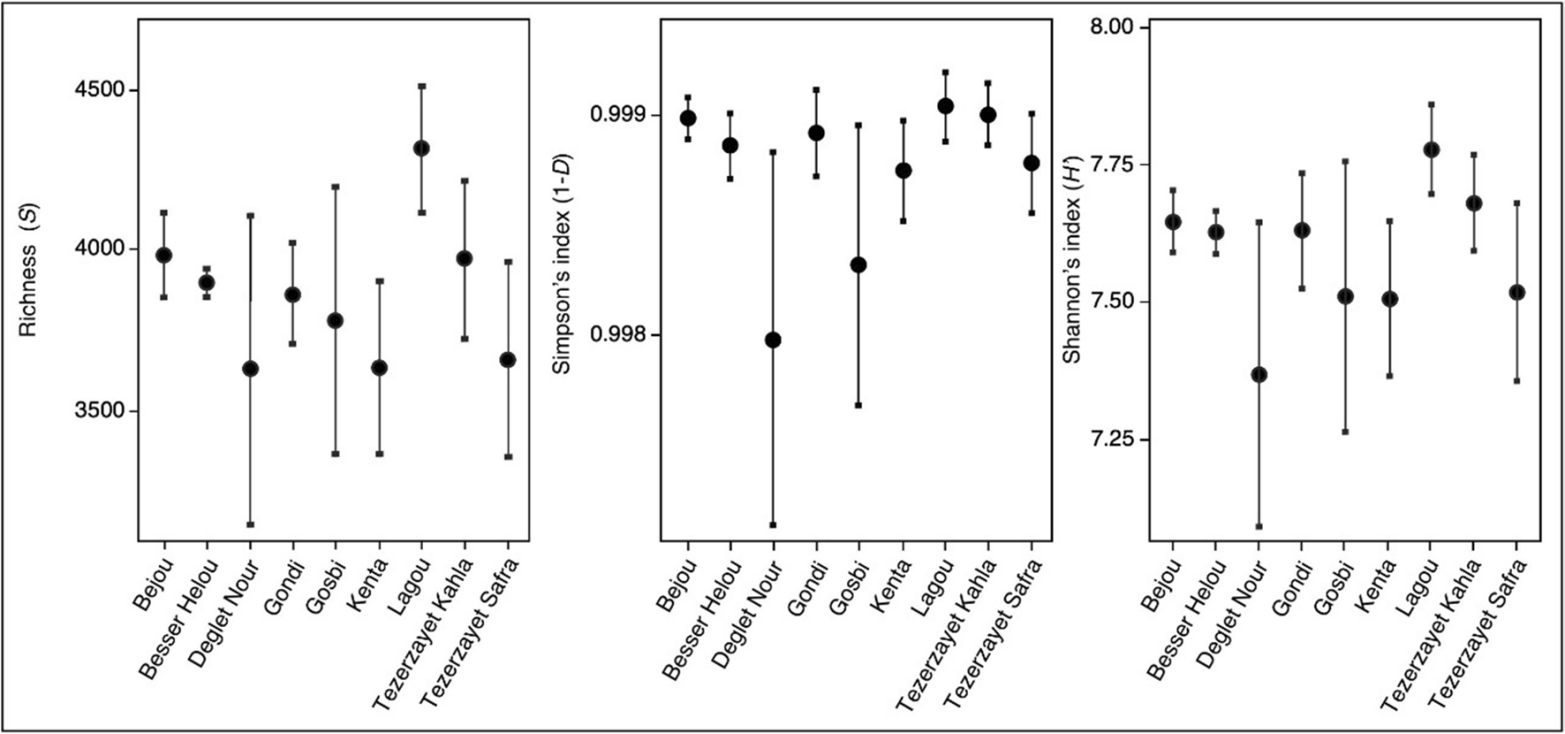
Diversity analysis of bacterial communities of the nine different date palm cultivars. Each plot graphically represents the richness of ASVs (*S*), Simpson (1-*D*), and Shannon (*H*′) indexes. There was no significant difference found among the cultivars for all three indexes

The bacterial community among different cultivars was compared by calculating rarefaction curves and by determining the richness (*S*) and alpha diversity indexes [Simpson (1-*D*) and Shannon (*H*′)]. Rarefaction curves revealed that the number of detected ASVs did not reach a plateau, suggesting that a higher sequencing depth could result in a better resolution (SI Fig. 3a, b). Although not significant, richness values were lowest in “Deglet Nour” and highest in “Lagou” cultivars (Fig. 1; SI Table 2). While no differences were detected for Simpson’s index (1-*D*; 0.99), a similar trend to richness values was observed for Shannon’s index indicating the lowest diversity in “Deglet Nour” and highest in “Lagou.”

**Fig. 2:**
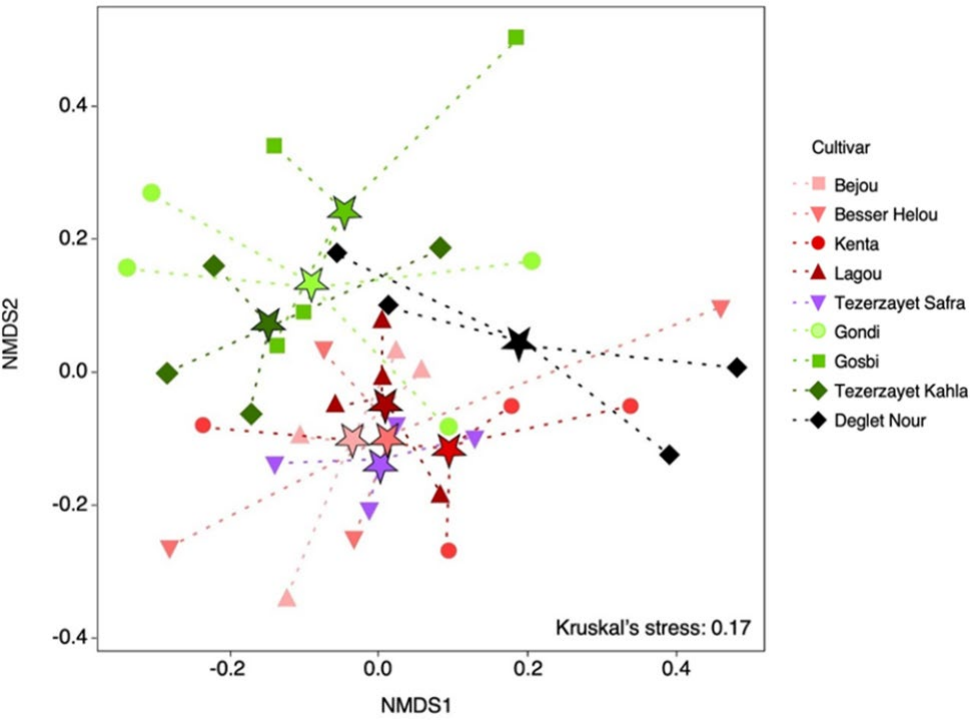
Non-metric multidimensional scale (NMDS) plots for bacterial communities found in the rhizospheric soils from different date palm cultivars. Clustering was performed using the Bray–Curtis dissimilarity. Each data point corresponds to a sample from a specific date palm cultivar, distinguished by color and shape as indicated in the legend. The centroid of each group is marked by a star in the color of the corresponding cultivar

For understanding similarities among bacterial communities from different samples, a non-metric multidimensional scaling (NMDS) analysis was conducted (Fig. 2). The NMDS analysis resulted in a well-represented cluster distribution, as indicated by a Kruskal's test *p*-value of 0.17. The ordination plot reveals the presence of two main clusters: a larger cluster comprising the microbial communities from “Bejou,” “Besser Helou,” “Kenta,” “Lagou,” and “Tezerzayet Safra” cultivars, and a separate cluster consisting of those from “Gosbi,” “Gondi,” and “Tezerzayet Kahla” cultivars. Lastly, “Deglet Nour” cultivar presented the most distinctive microbial community, being separated from the previous clusters.

The PERMANOVA analysis, based on the Bray–Curtis distance matrix (SI Table 3), revealed statistically significant results, suggesting that genotype differences account for 29% of the observed dissimilarities (*F* = 1.344, *R*^2^ = 0.285; *p* = 0.001). The cluster with the highest density of cultivar-associated microbial communities in the NMDS (“Bejou,” “Besser Helou,” “Kenta,” “Lagou,” and “Tezerzayet Safra”) globally displays less distinct bacterial communities, in particular among “Bejou,” “Besser Helou,” and “Lagou” cultivars. Therefore, the influence of genotypes in explaining dissimilarities among microbial communities is less pronounced and not significant (*F* = 1.09, *R*^2^ = 0.210, *p* = 0.232; PERMANOVA) among these cultivars. As for the second cultivar cluster (“Gondi,” “Gosbi,” and “Tezerzayet Kahla”), the “genotype” factor significantly accounts for the microbial dissimilarities observed among these cultivars (*F* = 1.210, *R*^2^ = 0.210, *p* = 0.006; PERMANOVA). However, bacterial communities within this cluster presented low dissimilarities among each other, except for the microbial communities of “Gondi” and “Tezerzayet Kahla” (SI Table 3). When comparing microbial communities between different cultivars from these two distinct clusters, in most cases the genotypes contribute significantly to the differences observed, emphasizing again the distinction between the two main groups. As previously described by the NMDS analysis, the “Deglet Nour” bacterial community is more distinct from the others, demonstrating significant dissimilarity when compared to the soil bacterial communities of “Tezerzayet Kahla,” “Bejou,” and “Besser Helou” (SI Table 3). Interestingly, despite “Tezerzayet” cultivars (“Kahla” and “Safra”) being clustered in different groups, they exhibit less distinct communities when compared to all cultivars (*F* = 1.124, *R*^2^ = 0.157, *p* = 0.02). The genetic diversity of date palm cultivars does not correlate with the bacterial diversity they harbor (*R* = 0.0623; *p* = 0.09; Mantel test). Additionally, PERMANOVA analysis showed no significant differences in bacterial communities among the different genetic clusters of date palms (SI Table 4).

### Richness and Diversity of Fungal Communities in Date Palm Soils

From the same 36 date palm soil samples, a higher number of raw reads (8,095,168) were obtained for the *ITS* barcode, ranging from 84,196 to 445,208 raw reads per sample (SI Table 5). After processing, approximately 4% of the total number of reads (136,119) were non-fungal ASVs, consisting of Viridiplantae (3%), unclassified sequences (0.4%), Metazoa (0.2%), and Choanoflagellates (0.1%). These non-fungal sequences were removed, resulting in a dataset of 2,964,985 high-quality fungal sequences. These sequences were then clustered into 6490 fungal ASVs, a reduced number of ASVs compared to the previously identified 13,194 bacterial ASVs. To ensure uniformity, subsampling of the fungal dataset was performed based on the sample with the lowest number of classified fungal sequences (22,688; *DEN4* sample). After subsampling, a total of 6442 fungal ASVs were assigned to 11 phyla, 39 classes, 81 orders, 168 families, and 250 genera.

**Fig. 3:**
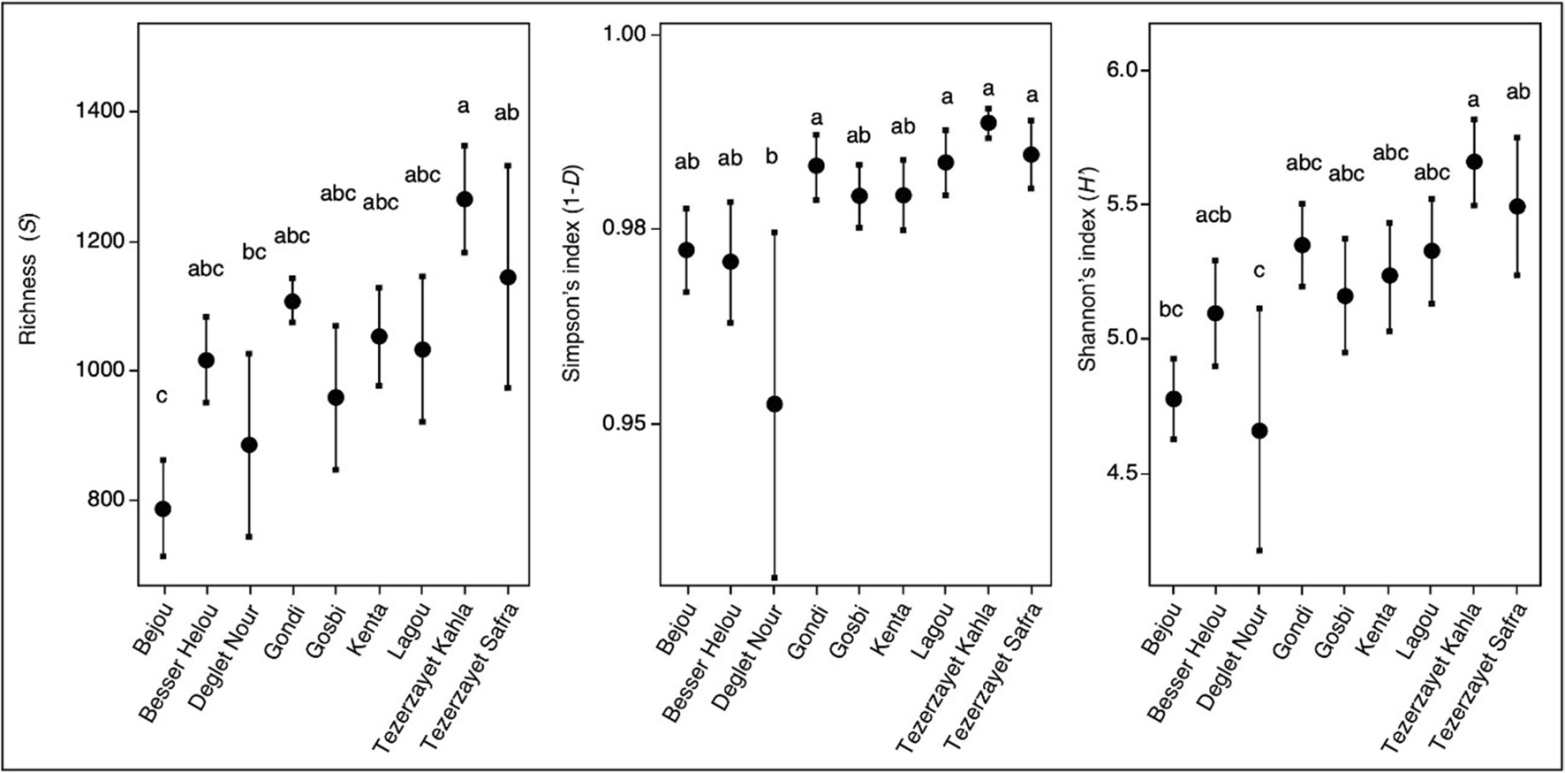
Diversity analysis for fungal communities of the nine different date palm cultivars. Each plot graphically represents the richness of ASVs (*S*), Simpson (1-*D*), and Shannon (*H*′) indexes. Different letters mean statistical differences, determined by ANOVA (*p* ≤ 0.05)

As previously performed with bacteria, fungal communities were characterized using rarefaction curves and measures of richness (*S*) and alpha diversities [Simpson’s index (1-*D*) and Shannon’s index (*H*′)]. Rarefaction curves provided a better representation of fungal communities compared to bacterial ones, but similarly, they did not reach a plateau suggesting the possibility of discovering additional ASVs through more in-depth sequencing (SI Fig. 4a, b). Both richness and diversity indexes indicated significant differences between the fungal communities associated with distinct cultivars (Fig. 3; SI Table 6). “Tezerzayet Kahla” exhibited fungal communities with the highest richness, which were closely followed by those from “Tezerzayet Safra.” In contrast, “Bejou” displayed fungal communities with the lowest richness, closely followed by “Deglet Nour.” A similar trend was observed in the analysis of diversity indexes, in which “Deglet Nour” consistently displayed the lowest diversity compared with “Tezerzayet” cultivars (“Kahla” and “Safra”) that consistently exhibited the highest diversity, setting them apart from the communities of “Deglet Nour.” Indeed, even though the bacterial findings lack statistical significance, the results indicate that “Deglet Nour” has lower bacterial and fungal diversity. The other cultivars demonstrate more diversity and compete to exhibit higher bacterial or fungal diversity.

**Fig. 4:**
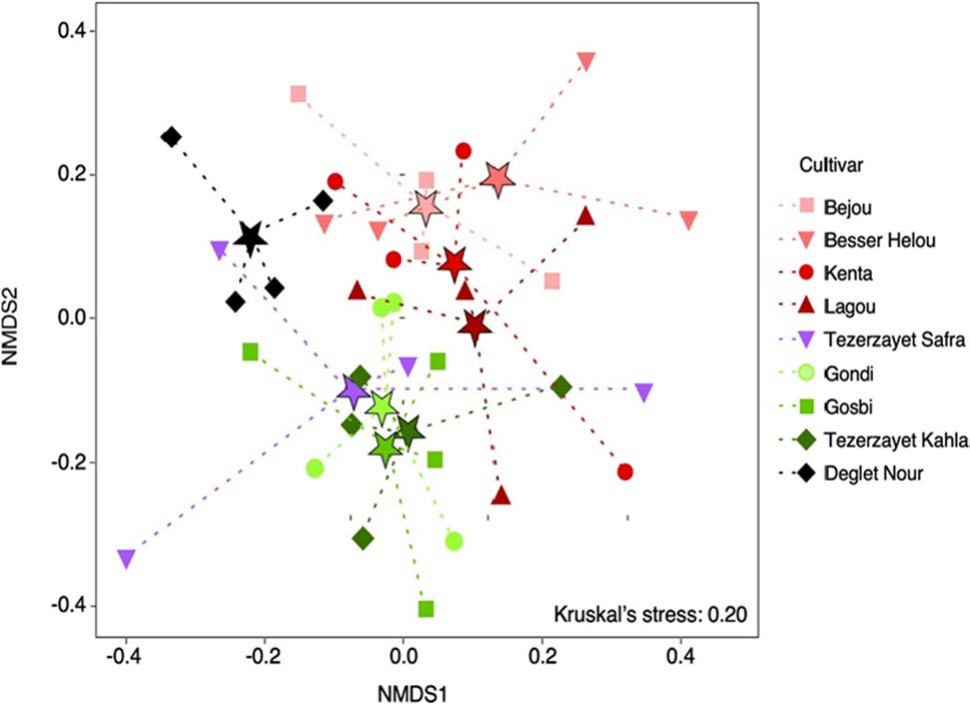
Non-metric multidimensional scale (NMDS) plot for fungal communities found in the rhizospheric soils from different date palm cultivars. Clustering was performed using the Bray–Curtis dissimilarity. Each data point corresponds to a sample from a specific date palm cultivar, distinguished by color and shape as indicated in the legend. The centroid of each group is marked by a star in the color of the corresponding cultivar

The NMDS analysis revealed a good representation of fungal community distribution, as confirmed by Kruskal’s stress test (*p* = 0.20, Fig. 4). The resulting plot revealed two distinguishable clusters: a first cluster composed of fungal communities associated to “Gosbi,” “Gondi,” “Tezerzayet Kahla,” and “Tezerzayet Safra” cultivars; and a second cluster consisting of communities associated with “Bejou,” “Besser Helou,” “Kenta,” and “Lagou” cultivars. Fungal communities from “Deglet Nour” appeared to be separated from the other cultivars. The PERMANOVA results highlighted the distinction of fungal communities among cultivars, revealing that 32% of the observed dissimilarities can be attributed to differences in genotypes (*F* = 1.595, *R*^2^ = 0.321; *p* = 0.001). In the second cluster (“Bejou,” “Besser Helou,” “Kenta,” and “Lagou”), the microbial communities are similar among them (*F* = 1.227, *R*^2^ = 0.235; *p* = 0.068), but some cultivars still exhibit significantly different communities (“Bejou” *vs.* “Lagou”; SI Table 7). However, the date palm cultivars found in the first cluster (“Gosbi,” “Gondi,” and “Tezerzayet Kahla”) significantly contribute to the differences found in corresponding fungal communities (*F* = 1.530, *R*^2^ = 0.251; *p* = 0.012). This is corroborated by the significant dissimilarities found between fungal communities of cultivars within the cluster (SI Table 7). The fungal community of “Deglet Nour” appears to be significantly distinct from all the other cultivars, with the only exception of *“*Tezerzayet Safra*”* and “Kenta” cultivars.

When examining the correlation between the genetic distances of different date palm cultivars and the Bray–Curtis distances of their corresponding fungal communities (*R* = 0.0467; *p* = 0.09; Mantel test), no significant correlation was found. However, within communities formed by the different genetic clusters identified, “Deglet Nour” stands out. Specifically, the fungal community of “Deglet Nour” genetic cluster differed significantly from all other cultivars, except from the one harbored by “Tezerzayet Safra” (*F* = 1.600, *R*^2^ = 0.210, *p* = 0.078; PERMANOVA; SI Table 8).

### Microbial Composition and Structure in Different Date Palm Cultivars

**Fig. 5:**
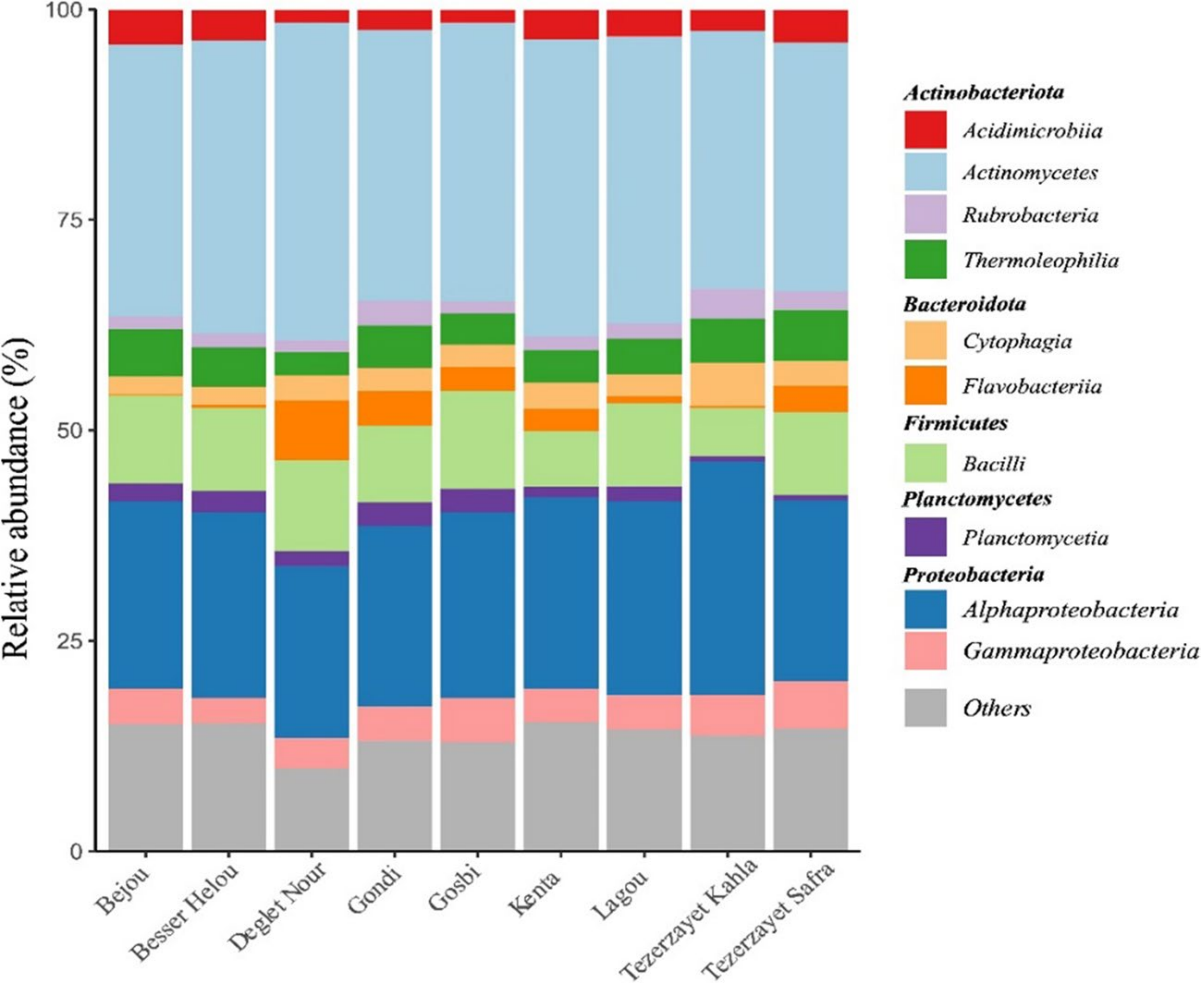
Structure of soil bacterial communities of different date palm cultivars. The relative abundance of the 10 most abundant classes is represented and the remaining classes are categorized as “others”

The taxonomic assignment of ASVs found in the soil under rhizospheric influence of the different date palm cultivars comprised 25 different bacterial phyla and 51 classes. The dominant phyla in all soil samples were *Actinobacteriota* (4%), *Proteobacteria* (27%), and *Firmicutes* (10%), followed by *Bacteroidota* (6%) and *Planctomycetes* (2%). To better understand the bacterial community composition, the top-10 most abundant classes were examined, with the remaining classes being classified as “others” (Fig. 5). The class *Actinomycetes* was highly represented in the soil of all date palm cultivars (average abundance of 34%), followed by *Alphaproteobacteria* (25%) and *Bacilli* (10%). These classes were indeed identified as date palm core microbiota based on the nine different cultivars (SI Fig. 5). Significant differences were observed among different cultivars in the *Alphaproteobacteria* class, as well as in less abundant classes like *Cytophagia* and *Planctomycetia* (SI Table 9).

**Fig. 6:**
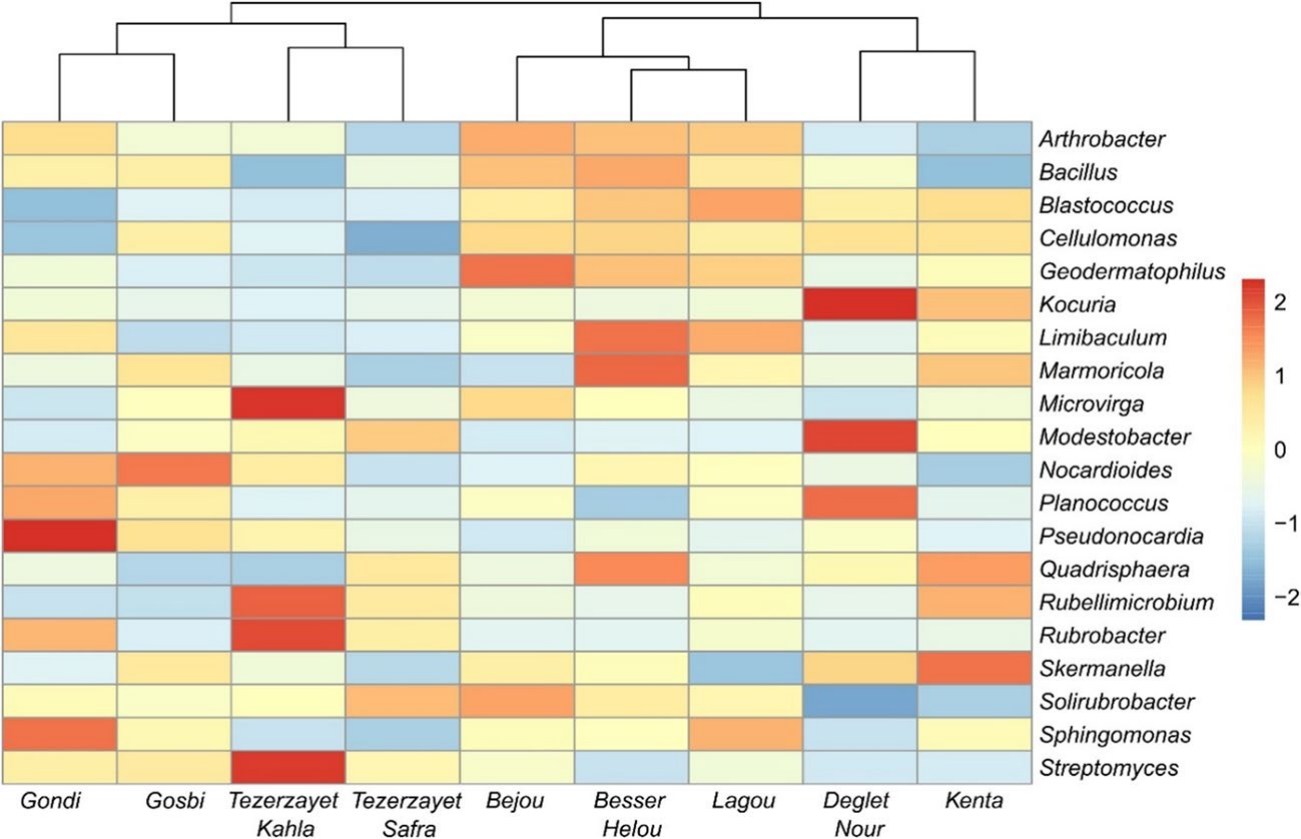
Heatmap analysis for characterization of bacterial communities at genus level of each date palm cultivar. Rows represents the relative percentage of each bacterial genus, and columns stands for different cultivars. The relative abundance for each bacterial genus was normalized and depicted by color intensity with the legend indicated on the right side of the figure

The most abundant bacterial genera present among cultivars were *Bacillus* (average abundance of 4%, *Bacilli* class), *Blastococcus* (4%, *Actinomycetes* class), and *Kocuria* (3%, *Actinomycetes* class), followed by *Geodermatophilus* (3%, *Actinomycetes* class), *Microvirga* (3%, *Alphaproteobacteria* class), and *Nocardioides* (3% *Actinomycetes* class) (SI Table 10; SI Fig. 6). The most distinctive aspect is the higher abundance of *Kocuria* genus in “Deglet Nour,” even though other significant differences were also detected among cultivars for *Geodermatophilus*, *Microvirga*, and *Nocardiodoides* genera. When analyzing the bacterial community at genus level, a heatmap analysis revealed the presence of two separate clusters (Fig. 6). One cluster aligns well with one of the NMDS clusters (Fig. 4) (“Bejou,” “Besser Helou,” and “Lagou” cultivars), being characterized by high abundances of *Arthtobacter*, *Bacillus*, *Blastoccous*, *Cellulomonas*, and *Geodermatophilus*. The other cultivar from this NMDS cluster (*Kenta*) also shows high abundance in *Blastococcus* and *Cellulomonas*, but contains a higher abundance of *Kocuria*, and *Modestobacter*, typical from “Deglet Nour.” This results in both cultivars clustering together. The remaining date palm cultivars (“Gondi,” “Gosbi,” “Tezerzayet Kahla,” and “Tezerzayet Safra”) were clustered together displaying variable genus abundance. Functional guild analysis of bacterial communities did not reveal any specific functions associated with plant growth-promoting rhizobacteria (PGPR) characteristics (SI Fig.7 ).

**Fig. 7:**
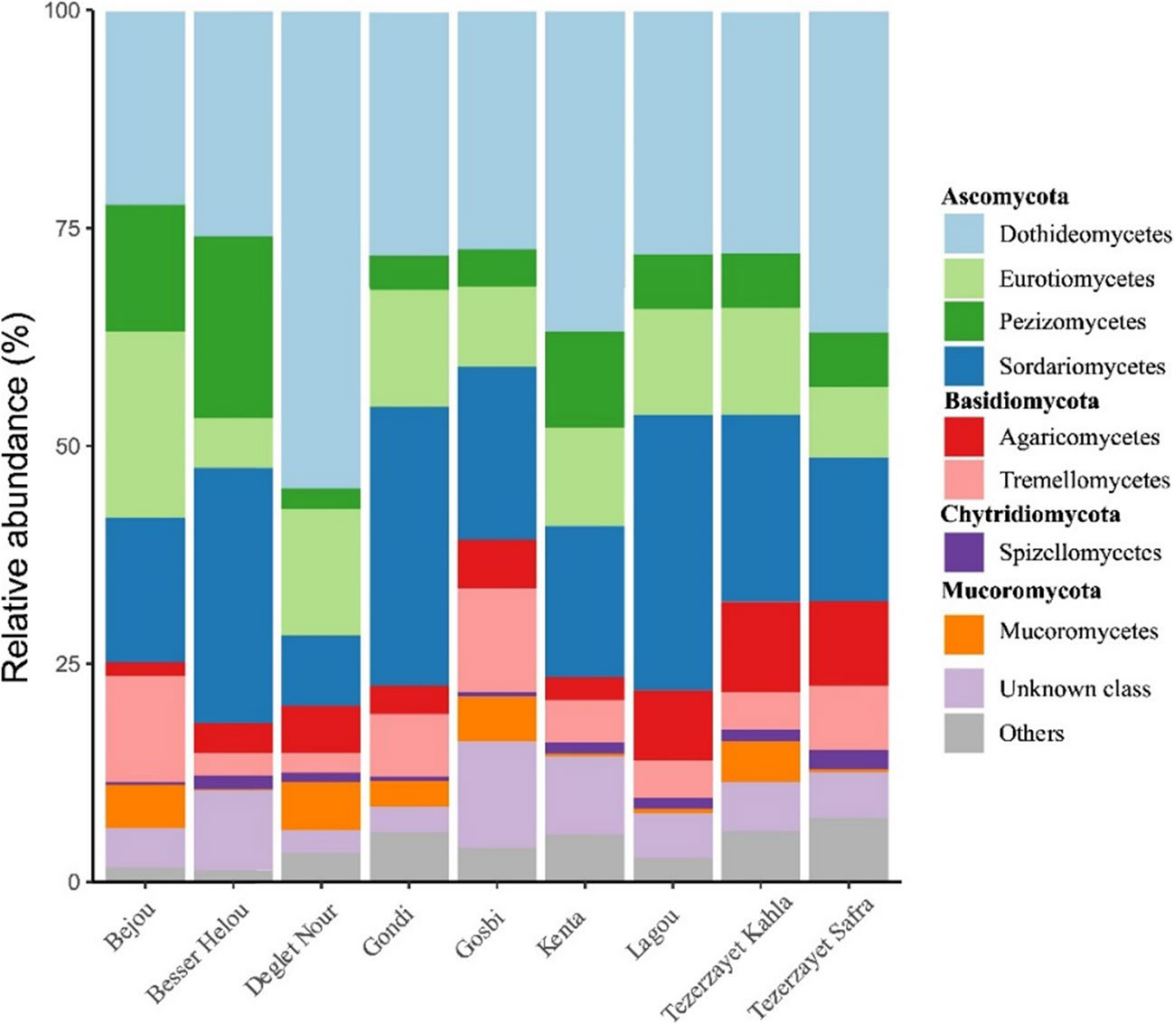
Structure of soil fungal communities of different date palm cultivars. The relative abundance of the 10 most abundant classes is represented and the remaining classes are categorized as “others”

The taxonomic assignment of ASVs for fungal communities identified 11 phyla and 39 classes. The Ascomycota phylum dominated (72% of fungal community), while the Basidiomycota only accounted for 11% (Fig. 7). The most abundant classes were Dothideomycetes (average abundance of 30%), followed by Sordariomycetes (21%), Eurotiomycetes (12%), and Pezizomycetes (8%) (Fig. 7, SI Table 11). Evident compositional differences were observed at class level among different date palm cultivars. Five out of the ten most abundant classes (Dothideomycetes, Eurotiomycetes, Pezizomycetes, Sordariomycetes, and Tremellomycetes) were found to be significantly dissimilar among the studied genotypes (SI Table 11). For example, Dothideomycetes ranged from 54.6% (“Deglet Nour”) to 22.2% (“Bejou”), and Sordariomycetes spanned from 32.5% (“Gondi”) to 8.1% (“Deglet Nour”). Based on the analysis of nine different cultivars, the main classes forming the core microbiome of date palms were Dothideomycetes, Sordariomycetes, and Eurotiomycetes (SI Fig. 8). Furthermore, changes in the bacterial communities present in the soil do not significantly affect the fungal ASVs. This finding was based on a co-occurrence network analysis, which found no patterns indicating a relationship or interaction between the bacterial and fungal communities.

**Fig. 8:**
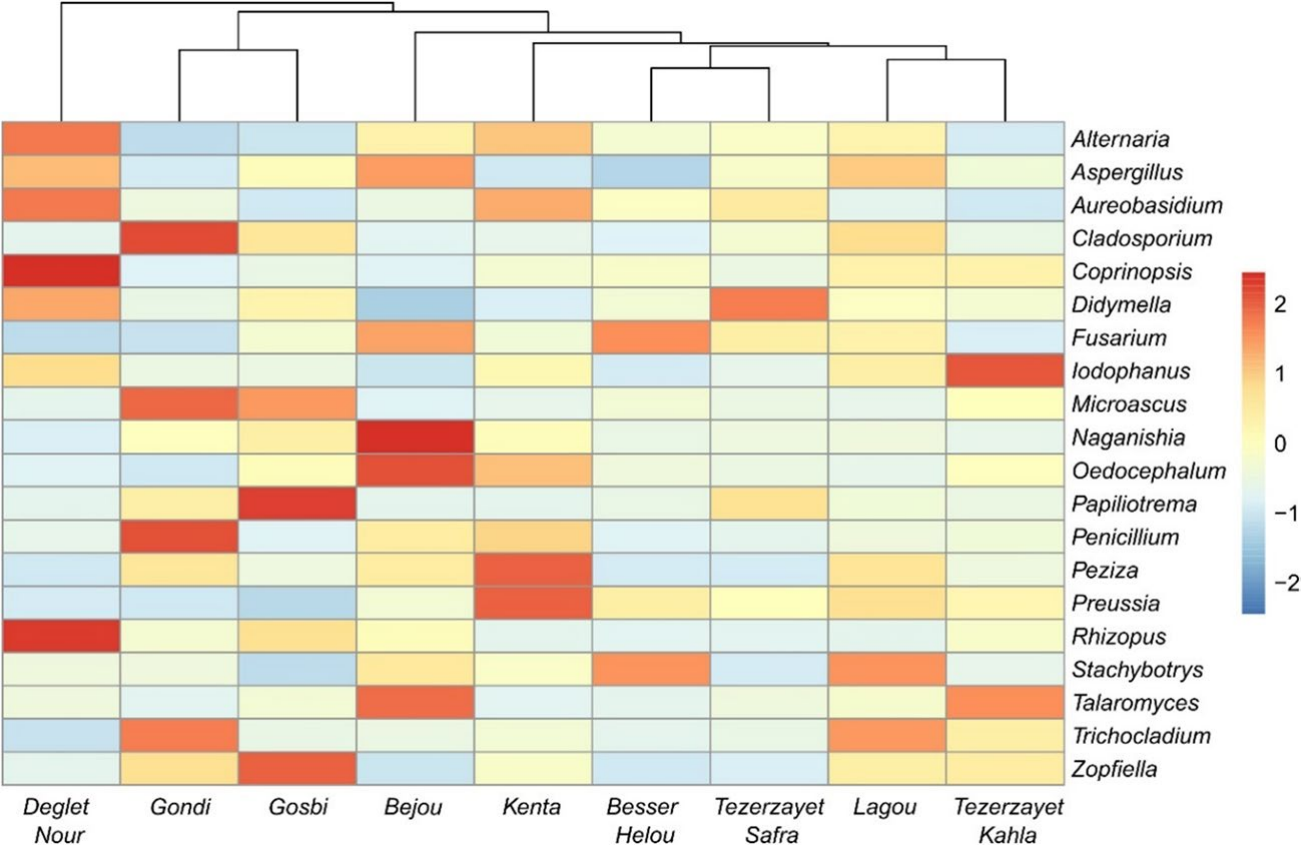
Heatmap analysis for characterization of fungal communities at genus level of each date palm cultivar. Rows represents the relative percentage of each fungal genus, and columns stands for different cultivars. The relative abundance for each fungal genus was normalized and depicted by color intensity with the legend indicated on the right side of the figure

The most prevalent fungal genera among the cultivars were *Aureobasidium* (10%, Dothideomycetes), *Alternaria* (6%, Dothideomycetes), *Trichocladium* (4%, Sordariomycetes), *Aspergillus* (3%, Eurotiomycetes), *Fusarium* (3%, Sordariomycetes), and *Naganishia* (3%, Tremellomycetes) (SI Table 12; SI Fig. 9). Other dominant genera display significant variations across different cultivars. Indeed, among the six more abundant genera, *Alternaria*, *Trichocladium*, *Aspergillus*, and *Naganishia* revealed significant differences among cultivars (as depicted in SI Table 12). The heatmap analysis based on fungal genera revealed “Deglet Nour” as an outlier cultivar, displaying *Coprinopsis*, *Rhizopus, Alternaria*, *Aureobasidium*, and *Aspergillus*, as the prevalent genera (Fig. 8). Among the remaining cultivars, “Gondi” and “Gosbi” form a distinct cluster, distinguished by *Cladosporium*, *Microascus*, *Papiliotrema*, and *Zopfiella*. Upon analyzing the functional guild of fungal communities associated with different cultivars, a clear distinction was observed in “Deglet Nour,” which had a higher proportion of the community associated with pathogenic functions (SI Fig. 10).

### Microbial Communities Relate to Tolerance of Biotic and Abiotic Stress

The studied cultivars were categorized by date palm farmers into three tolerance groups based on their ability to withstand salinity conditions. The groups comprised high, medium, and low tolerant cultivars. The most resilient cultivars include “Besser Helou” (from genetic cluster 3), “Bejou,” and “Kenta” (both from genetic cluster 4), followed by the group comprising “Gondi,” “Gosbi,” and “Lagou” (from genetic cluster 4), “Tezerzayet Kahla” (cluster 1), “Tezerzayet Safra” (cluster 0), and lastly “Deglet Nour” (cluster 2) identified as the most sensitive cultivar. The NMDS analysis revealed three distinct clusters in both microbial communities, corresponding to previously described tolerance levels for each cultivar (SI Fig. 11a, b). The fungal communities had more distinctive clusters than the bacterial communities.

## Discussion

In recent years, microbial communities associated with plants have gained significant interest due to their role in improving plant fitness against abiotic and biotic stresses. The primary goal of this work was to explore the microbial diversity in soils influenced by date palms (*Phoenix dactylifera*) root systems and to determine whether different genotypes could lead to distinct microbial compositions. Given that the date palms sampled in this study were growing within the same oasis, sharing identical soil characteristics and cultural management practices, we hypothesized that variations in microbiota within the soil influenced by roots were primarily attributed to disparities in genotype-specific variations.

The results revealed a highly diverse and enriched microbial community in date palm soils, in which 13,189 bacterial and 6442 fungal ASVs were identified. Only 80% of bacterial and 64% of fungal ASVs were classified up to the family level. A classification to the genus level was only achieved for 52% (bacteria) and 64% (fungi) ASVs, respectively. Furthermore, a small fraction of ASVs (0.1% for bacteria and 0.4% for fungi datasets) were not classified. These results confirm the widely acknowledged fact that there is still much soil diversity that remains to be fully characterized [51]. The taxonomic assignment of ASVs provided insights into the dominant phyla, classes and genera within both bacterial and fungal communities. Bacterial communities were dominated by *Actinobacteriota*, *Proteobacteria*, and *Bacteroidota*, while fungal communities were primarily dominated by Ascomycota. Furthermore, the core microbiota in the soil influenced by date palm roots mainly consisted on *Actinobacteria*, *Alphaproteobacteria*, and *Bacilli* (bacteria) and on Dothideomycetes, Sordariomycetes, and Eurotiomycetes (fungi). These results are in agreement with other studies where Ascomycota was also found to dominate the rhizospheres of seedlings of two distinct date palm cultivars (“Umsila” and “Zabad”) from Oman [21]. Comparative studies with proximal soils around the roots of the same date palm cultivars [24], as well as rhizospheric soils from Qatari date palm farms [25], revealed a similar overrepresentation of *Actinobacteria* and specific classes like *Alphaproteobacteria*. This indicates specific microbial preferences associated with date palm soils. In contrast, the root system of the “Deglet Nour” date palm cultivar in Tunisia was reported to be associated with bacterial communities dominated by *Gammaproteobacteria*, primarily *Enterobacteriales* [52]. This suggests a selective process by the date palm roots that favors a conserved core microbiome, which is capable of providing essential plant growth-promoting traits. Notably, distinct bacterial diversity and community structures have been observed in date palm roots and surrounding soils, where factors such as water availability and soil chemistry significantly influence these communities [22].

In the present work, the microbial communities present in the influential region of date palm roots exhibited substantial microbial richness and diversity, with bacterial communities surpassing their fungal counterparts. Despite this, no significant differences were observed in bacterial communities among cultivars, in contrast to fungal communities, where noteworthy distinctions on microbial richness and diversity emerged between genotypes. Previous studies have reported similar findings regarding date palm rhizosphere [25, 53]. While bacterial communities were reported to be less affected by different *P. dactylifera* genotypes and were highly correlated with geographic location, there was a stronger effect of cultivars on fungal community modulation.

The analysis of microbial communities across all genotypes highlighted distinct dynamics between bacterial and fungal populations in the studied soils. Interestingly, two distinct clusters emerged in both bacterial and fungal assemblies, with specific date palm cultivars consistently explaining the observed distribution patterns of both microbial communities. The date palm cultivars “Gondi,” “Gosbi,” and “Tezerzayet Kahla” clustered differently from the group comprising “Bejou,” “Besser Helou,” “Kenta,” and “Lagou,” in both microbial communities. This suggests that the recruitment of microbes within each group may share similarities. This finding aligns with the increasing recognition of the role played by plant genotypes in shaping soil microbial communities and is supported by statistical analysis indicating that the genotype of the date palm has a significant influence over the microbial communities. A possible explanation may lie in rhizodeposition, which allows plants to select microbes from bulk soil. This process varies with the exudates expelled by distinct plant species or genotypes. Therefore, the clustering of these cultivars may suggest a resemblance to their rhizodeposition profile. However, when using genetic data from the date palm cultivars included in this study, there was lack of a significant overall correlation between genetic distance and corresponding microbial community similarity distances. This finding suggests that genetically similar cultivars do not consistently exhibit more similar microbial community profiles compared to genetically dissimilar ones. However, significant distinctions were noted when analyzing genetic groups instead of individual cultivars. In particular, the “Deglet Nour” cultivar, which forms a distinct genetic group, showed a significant difference in the fungal communities compared to other genetic groups. This suggests a distinct microbiota assembly, potentially influenced by genetic erosion when this cultivar is grown in a monoculture within the oasis [15]. Additionally, the genetic group “Tezerzayet Safra” exhibited the most common fungal community profile when considering genetic distance relative to other cultivars. As there was no correlation between the relative position of the sampled date palms on the land plot and the clusters formed in the NMDS. Reinforcing the hypothesis of genotype-specific pressure for shaping microbial communities in “Deglet Nour.” Additionally, it was observed that fungal communities were more influenced by the date palm genotype than bacterial communities, as evidenced by the higher percentage of dissimilarity explained by the genotype factor. Similar results on the genotype effect on fungal assembling have been previously reported for *P. dactylifera* [53] and other species like *Pinus radiata* [54] and *Arabidopsis thaliana* [55]. This contrasts with other studies that report no role of plant genotype in determining the fungal community structure in date palms [21]. Using “Umsila” and “Zabad” date palm cultivars, significant changes in fungal communities were observed in the epiphytic root only under high salinity stress (250 mM salt solutions) [21], while bacterial communities did not show significant cultivar changes in either stressed or unstressed date palms [24]. In accordance, our findings on the insignificant changes of bacterial communities with different cultivars contrast with studies suggesting that the genotype, rather than soil type, determines bacterial community assembly in root systems [21, 24, 25]. All these differences highlight the differential responses of bacterial and fungal communities to both genetic and environmental factors in date palm, indicating that environmental stress factors, such as salinity, could play a role in shaping fungal communities [21, 23].

In this study, we explore the potential correlation between date palm tolerance to abiotic and biotic stresses and variations observed in microbial communities. The categorization of date palm cultivars into high, medium, and low tolerance groups aligns with the findings from the NMDS analysis, depicting distinct microbiota clusters from different date palm cultivars. This may suggest a possible link between genotypes, stress resistance, and the assembly of microbial communities, particularly evident in the analysis of fungal communities. Previous studies have highlighted this connection, showing that date palm seedlings from different cultivars exposed to salinity conditions exhibit contrasting fungal communities [21], but not bacterial communities [24]. The authors suggested that salinity exerts a more pronounced influence on fungal community composition than plant genotype, but emphasize the importance of plant genomic makeup in deciphering salinity tolerance mechanisms [21]. However, soil bacterial communities associated with date palms could serve as potential “seed sources” for recruitment by stressed date palm roots. For example, saline groundwater irrigation has been reported to change the bacterial communities of bulk soil associated to date palm by selectively enriching for salt-tolerant bacteria that are capable of adapting to salinity stress [23]. Indeed, it is now well established that microorganisms are recruited from the surrounding soil through the release of exudates from roots [56]. Differences in plant genotype would result in different rhizodeposits, which could lead to distinct microbial community structures [57]. Although our findings suggest a potential correlation between date palm genotypes and microbial community structure, we recognize that this relationship remains speculative and requires further experimental validation. Future research should focus on directly investigating root-associated microbial communities under natural but contrasting environmental conditions, while controlling for soil characteristics, to better isolate the effects of date palm genotype on microbial community assembly.

The functional analysis did not reveal any bacterial traits that could distinguish date palm genotypes, but there was a differential predominance of fungal guilds. Among these, saprotrophs were found to be the most abundant trophic mode. A significant finding was the presence of fungal taxa with pathogenic features in “Deglet Nour,” aligning with the observation that this genotype was highly enriched in *Alternaria* genus, known for containing some of the most destructive plant pathogens [58]. Another interesting result is the presence of ectomycorrhizal fungi on soils from the most salt-tolerant cultivars (“Bejou,” “Besser Helou,” and “Kenta”). While numerous studies emphasize the importance of arbuscular fungi for the sustainability of date palm trees (e.g., [59]), less attention has been given to their capacity to form ectomycorrhiza [60]. Fungal community analysis revealed a higher prevalence of ectomycorrhizal fungi (EcM) in contrast to arbuscular fungi, particularly on salt-stress tolerant cultivars. Plants engaging with EcM exhibit better resistance to biotic stress [61] and can relieve abiotic stresses, such as drought [62]. Therefore, the higher predominance of ectomycorrhizal fungi in salt-stress-tolerant cultivars suggests a potential role in enhancing tolerance to both biotic and abiotic stresses. Further investigation is needed to fully understand the potential of specific EcM species in enhancing date palm tolerance.

In conclusion, this study sheds light on the impact of date palm genotype on soil microbial communities associated with roots. Despite being grown under identical environmental conditions, distinct microbial assemblies, mainly fungal communities, were observed among different cultivars, suggesting genotype-specific interactions. While the influence of soil characteristics should be considered, our findings highlight the significant role that plant genotype plays in shaping these microbial communities, mainly the fungal community. Furthermore, our findings suggest potential correlations between date palm genotype, stress tolerance, and microbial community assembly, which was particularly evident in fungal communities. The presence of pathogenic fungi in “Deglet Nour” and ectomycorrhizal fungi in salt-tolerant cultivars highlights the importance of genotype-specific microbial interactions in plant resilience. These findings suggest a potential link between microbial assemblies and the traditional knowledge regarding cultivar salt stress tolerance, although further laboratory validation is required. At the end, this study contributes to the understanding of date palm-associated microbial dynamics, emphasizing the need to understand the mechanisms underlying genotype-specific microbial interactions and their impact on plant health and stress tolerance.

## Competing Interests

The authors declare no competing interests.

### Supplementary Information

Below is the link to the electronic supplementary material.

Supplementary file1 (DOCX 3584 KB)

## Data Availability

The datasets generated during the current study are available in the NCBI repository under BioProject accession PRJNA1109279.
