# Supplementary material for: Exploring the Influence of Date Palm Cultivars on Soil Microbiota
Source: Microb Ecol. 2024 Aug 1;87(1):103. doi: 10.1007/s00248-024-02415-x (PMC11294395; doi:10.1007/s00248-024-02415-x)
Supplement: Supplementary file 1 — Supplementary file1 (DOCX 3584 KB) [file 248_2024_2415_MOESM1_ESM.docx]

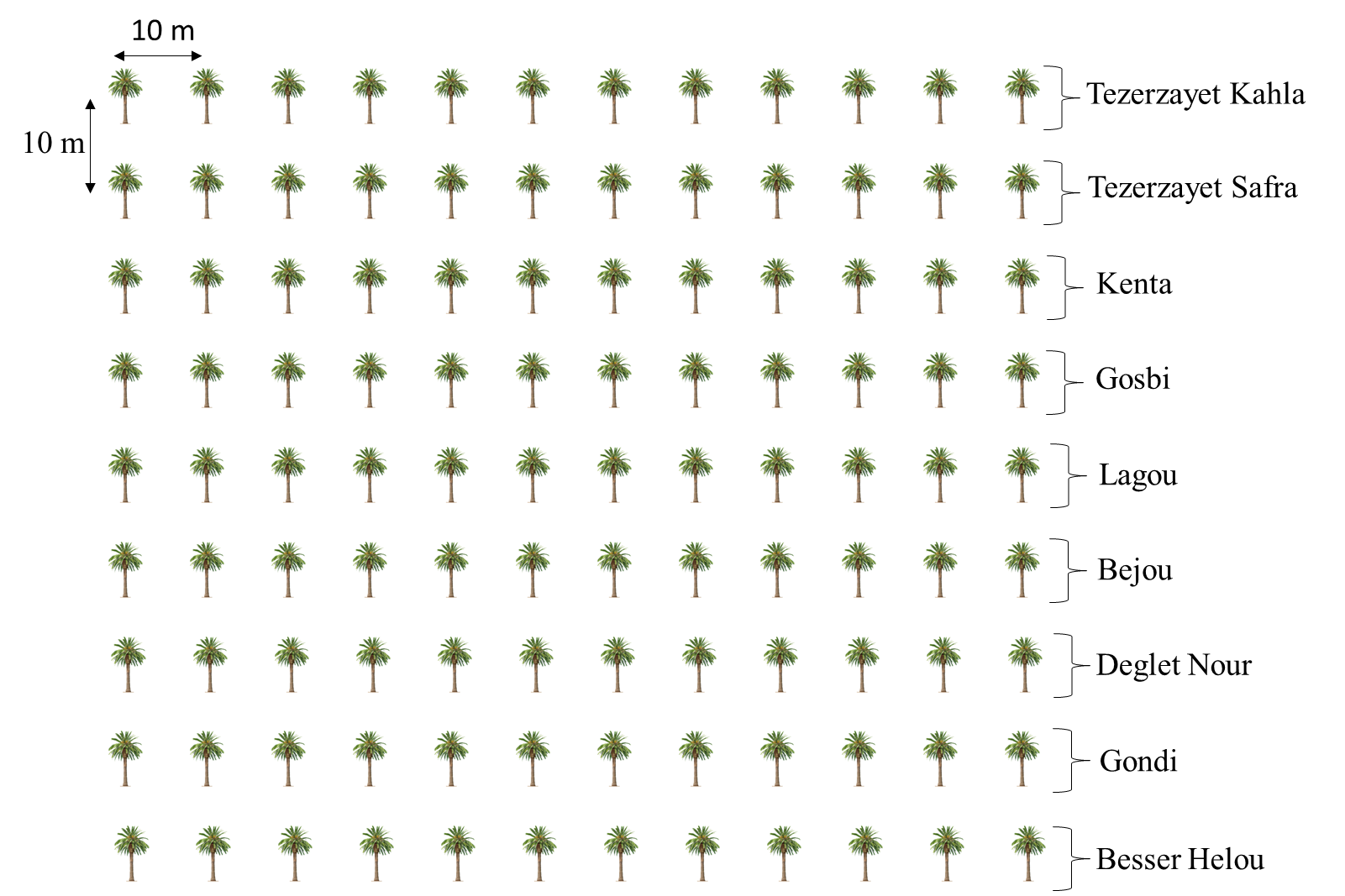


**SI Fig. 1** The sampled cultivars were located within a plot in the Tozeur region of Tunisia. Each cultivar was planted in a specific row containing 12 trees, with approximately 10 meters between each tree. Image generated by BioRender (<https://www.biorender.com/> ).


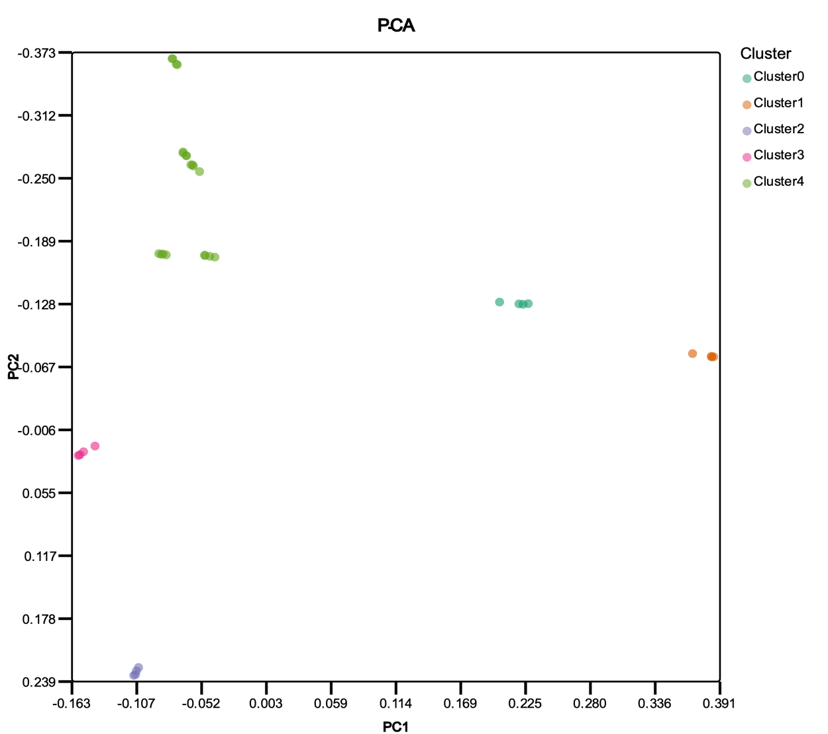

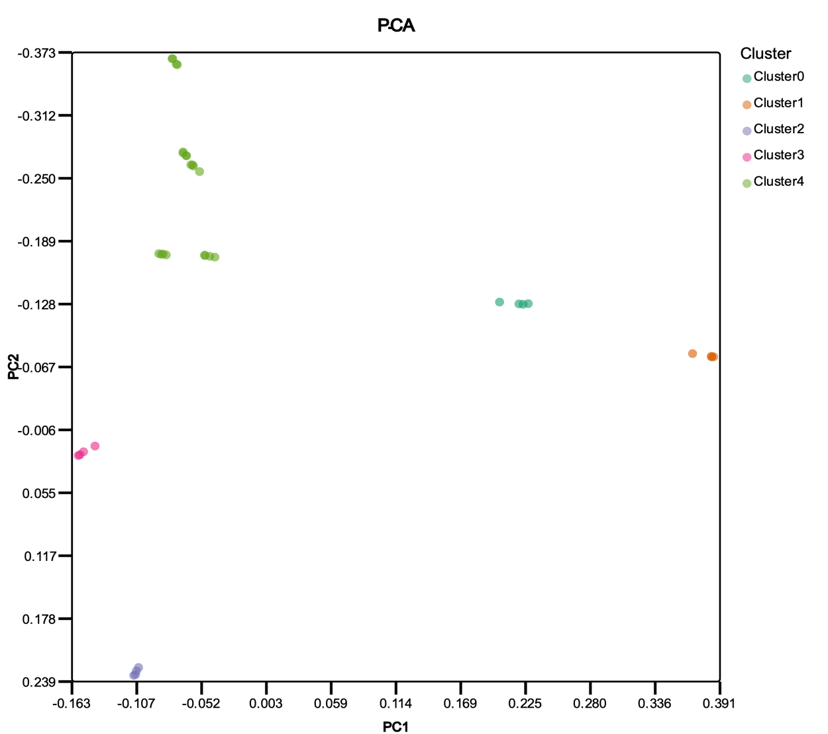


**SI Fig. 2** Principal component analysis (PCA) for cultivar genetic analysis. Date palm samples were clustered in 5 groups: Cluster 0 (‘Tezerzayet Safra’), Cluster1 (variety ‘Tezerzayet Kahla’), Cluster2 (‘Deglet Nour’) Cluster3 (‘Besser Hellou’); Cluster4 (‘Gondi’, ‘Gosbi’, ‘Lagou’, ‘Kenta’, ‘Bejou’). The first principal component (PC1) explained 12.00% of the total variance and clearly separated different clusters. The second principal component (PC2) accounted for 11.05% of the total variance and also contributed to separating clusters.

**SI Table 1** Number of reads obtained by *Illumina MiSeq* metabarcoding of *16S* DNA samples, obtained from soils of nine different date palm cultivars. The raw dataset was processed as described in the Materials and Methods section. Classified sequences correspond to those identified as bacterial before dataset subsampling.

| **Cultivar** | **Samples** | **Raw read** | **Merged**  **reads** | **Processed**  **reads** | **Classified**  **sequences** |
| --- | --- | --- | --- | --- | --- |
| *Bejou* | BEJ1 | 145,840 | 66,291 | 37,150 | 37,142 |
|  | BEJ2 | 176,855 | 80,389 | 43,825 | 43,814 |
|  | BEJ3 | 139,427 | 63,376 | 34,598 | 34,576 |
|  | BEJ4 | 132,162 | 60,074 | 35,014 | 35,008 |
|  | **Total** | **594,284** | **270,130** | **150,587** | **150,540** |
| *Besser*  *Helou* | BEH1 | 104,957 | 47,708 | 25,974 | 25,958 |
|  | BEH2 | 134,677 | 61,217 | 35,353 | 35,340 |
|  | BEH3 | 116,336 | 52,880 | 28,051 | 28,034 |
|  | BEH4 | 105,432 | 47,924 | 24,026 | 24,003 |
|  | **Total** | **461,402** | **209,729** | **113,404** | **113,335** |
| *Deglet*  *Nour* | DEN1 | 116,714 | 53,052 | 33,004 | 33,000 |
|  | DEN2 | 220,446 | 100,203 | 55,437 | 55,428 |
|  | DEN3 | 105,721 | 48,055 | 26,911 | 26,910 |
|  | DEN4 | 120,989 | 54,995 | 28,952 | 28,946 |
|  | **Total** | **563,870** | **256,305** | **144,304** | **144,284** |
| *Gondi* | GND1 | 115,051 | 52,296 | 25,609 | 25,609 |
|  | GND2 | 98,617 | 44,826 | 23,064 | 23,064 |
|  | GND3 | 74,278 | 33,763 | 18,025 | 18,012 |
|  | GND3 | 100,845 | 45,839 | 27,480 | 27,480 |
|  | **Total** | **388791** | **176,724** | **94,178** | **94,165** |
| *Gosbi* | GSB1 | 97,754 | 44,434 | 24,026 | 24,024 |
|  | GSB2 | 120,678 | 54,854 | 30,696 | 30,693 |
|  | GSB3 | 67,364 | 30,620 | 16,993 | 16,991 |
|  | GSN4 | 134,508 | 61,140 | 27,691 | 27,689 |
|  | **Total** | **420,304** | **191,048** | **99,406** | **99,397** |
| *Kenta* | KT1 | 80,192 | 36,451 | 14,618 | 14,549 |
|  | KT2 | 142,324 | 64,693 | 28,436 | 28,322 |
|  | KT3 | 136,461 | 62,028 | 37,886 | 37,879 |
|  | KT4 | 149,736 | 68,062 | 42,005 | 42,003 |
|  | **Total** | **508,713** | **231,234** | **122,945** | **122,795** |
| *Lagou* | LAG1 | 137,704 | 62,593 | 34,538 | 34,530 |
|  | LAG2 | 108,717 | 49,417 | 27,956 | 27,948 |
|  | LAG3 | 118,989 | 54,086 | 28,577 | 28,561 |
|  | LAG4 | 167,208 | 76,004 | 44,053 | 44,052 |
|  | **Total** | **532,618** | **242,100** | **135,124** | **135,091** |
| *Tezerzayet* *Kahla* | TZK1 | 146,097 | 66,408 | 31,438 | 31,419 |
|  | TZK2 | 182,846 | 83,112 | 38,061 | 38,042 |
|  | TZK3 | 147,285 | 66,948 | 29,038 | 29,010 |
|  | TZK4 | 139,288 | 63,313 | 27,480 | 27,013 |
|  | **Total** | **615,516** | **279,781** | **126,017** | **125,484** |
| *Tezerzayet*  *Safra* | TZS1 | 179,720 | 81,691 | 32,993 | 32,993 |
|  | TZS2 | 153,274 | 69,670 | 32,201 | 32,185 |
|  | TZS3 | 120,082 | 54,583 | 25,012 | 24,992 |
|  | TZS4 | 143,884 | 65,402 | 30,665 | 30,580 |
|  | **Total** | **596,960** | **271,346** | **120,871** | **120,750** |
| **Total** | | **4,682,458** | **2,128,397** | **1,106,836** | **1,105,841** |

**a**

**b**


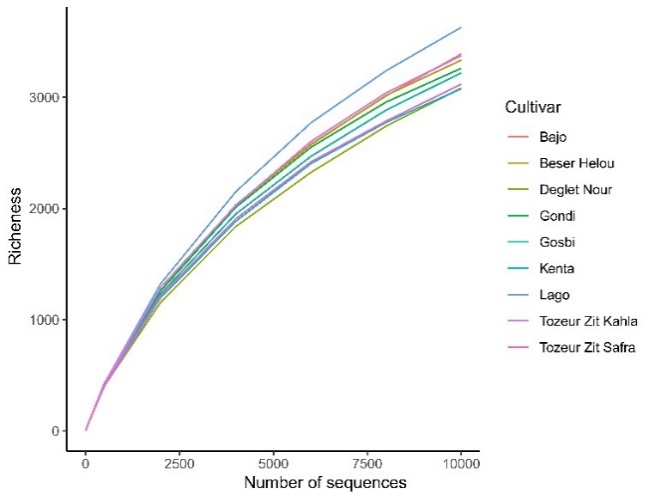

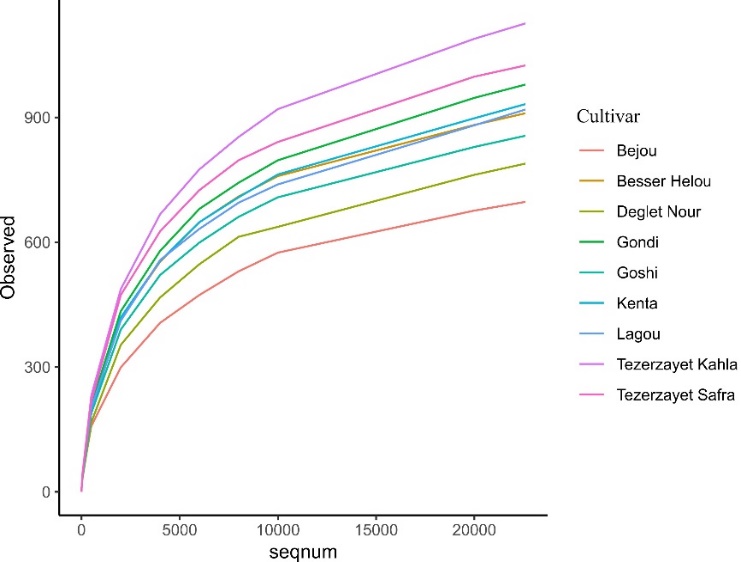

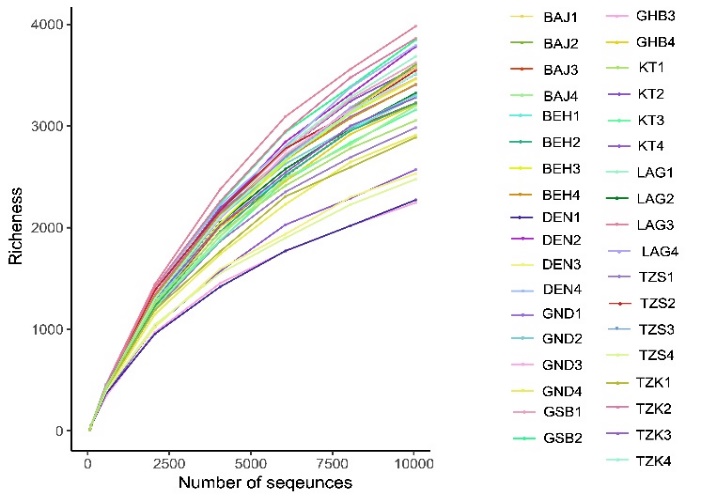

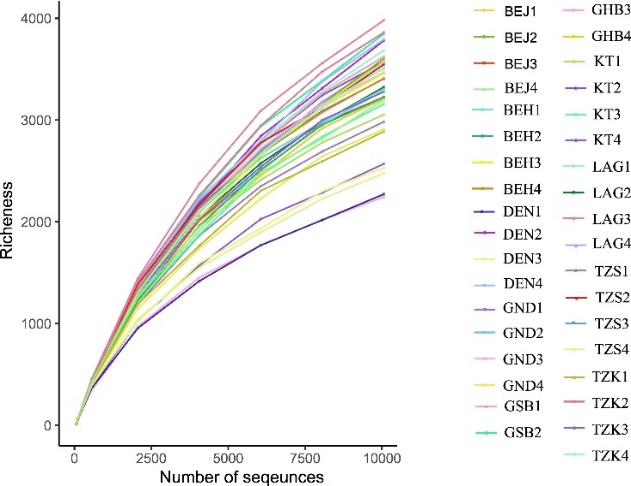


**SI Fig. 3** Rarefaction curves illustrating the diversity of bacterial communities, present in each date palm cultivar (a) and across distinct soil samples (b).

**SI Table 2** Richness (*S*) and alpha diversity indexes [Simpson’s index (1-*D*) and Shannon index (*H*’)] of bacterial communities found in the soil of different date palm cultivars. Results are displayed as the mean value ± standard deviation (SD). No statistical differences were found among cultivars. The values highlighted in bold represent the lowest values for richness and diversity, while those presented in bold and italics represent the highest values.

| **Cultivar** | **Richness (*S*)** | **Simpson’s index**  **(1-*D*)** | **Shannon’s index (*H*’)** |
| --- | --- | --- | --- |
| ***Bejou*** | 3,977 ± 265 | 0.99 ± 0.187 | 7.45 ± 1.14e^-04^ |
| ***Besser Helou*** | 3,888 ± 849 | 0.99 ± 2.93e^-04^ | 7.62 ± 7.76e^-02^ |
| ***Deglet Nour*** | **3,613 ± 972** | 0.99 ± 1.69e^-0.3^ | **7.36 ± 5.52e^-03^** |
| ***Gondi*** | 3,852 ± 312 | 0.99 ± 3.90e^-04^ | 7.62 ± 2.09e^-04^ |
| ***Gosbi*** | 3,771 ± 846 | 0.99 ± 1.27e^-03^ | 7.50 ± 0.487 |
| ***Kenta*** | 3,622 ± 542 | 0.99 ± 4.56e^-04^ | 7.50 ± 0.284 |
| ***Lagou*** | ***4,314 ± 392*** | 0.99 ± 3.15e^-04^ | ***7.77 ± 0.161*** |
| ***Tezer. Kahla*** | 3,964 ± 492 | 0.99 ± 2.74e^-04^ | 7.67 ± 0.175 |
| ***Tezer. Safra*** | 3,645 ± 617 | 0.99 ± 4.52e^-04^ | 7.51 ± 0.322 |

**SI Table 3** Permutational analysis of variance (PERMANOVA) of bacterial communities found in date palm soil samples from each cultivar, using Bray-Curtis. Both *F* and *R^2^* (in bold) values are represented. Dissimilarities found among cultivars from ‘Bejou’, ‘Besser Helou’, ‘Kenta’, ‘Lagou’, and ‘Tezerzayet Safra’ cluster are highlighted in light pink and those from ‘Gosbi’, ‘Gondi’, and ‘Tezerzayet Kahla’ cluster in green. Statistical significance is denoted by an asterisk and was considered for *p* < 0.05.

|  | ***Bejou*** | ***Besser Helou*** | ***Deglet Nour*** | ***Gondi*** | ***Gosbi*** | ***Kenta*** | ***Lagou*** | ***Tezer. Kahla*** | ***Tezer. Safra*** |
| --- | --- | --- | --- | --- | --- | --- | --- | --- | --- |
| ***Bejou*** |  | **0.1377** | **0.2204** | **0.2171** | **0.2051** | **0.1792** | **0.1325** | **0.1957** | **0.1680** |
| ***Besser Helou*** | 0.9580 |  | **0.2057** | **02106** | **0.2133** | **0.1481** | **0.1543** | **0.2170** | **0.1555** |
| ***Deglet Nour*** | 1.6964* | 1.5545* |  | **0.1624** | **0.1798** | **0.1658** | **0.1882** | **0.2235** | **0.1705** |
| ***Gondi*** | 1.6644* | 1.6003 | 1.1634 |  | **0.1480** | **0.2148** | **0.1824** | **0.1942** | **0.1818** |
| ***Gosbi*** | 1.5484* | 1.6271* | 1.3153 | 1.0420 |  | **0.2170** | **0.1942** | **0.1640** | **0.1828** |
| ***Kenta*** | 1.3105 | 1.0486 | 1.1925 | 1.6416 | 1.6902 |  | **0.1678** | **0.2028** | **0.1290** |
| ***Lagou*** | 0.91655 | 1.0946 | 1.3905 | 1.3474 | 1.4465* | 1.2096 |  | **0.1972** | **0.1711** |
| ***Tezer. Kahla*** | 1.4597 | 1.6631* | 1.7274* | 1.4457* | 1.1773 | 1.5266 | 1.4742 |  | **0.1579** |
| ***Tezer. Safra*** | 1.2112 | 1.1049 | 1.2335 | 1.3329 | 1.3422* | 0.8884 | 1.2386 | 1.1247 |  |

**SI Table 4** Permutational analysis of variance (PERMANOVA) of bacterial communities found in the different genetic clusters formed by the different date palm cultivars, using Bray-Curtis dissimilarities. Both *F* and *R*^2^ (in bold) values are represented. No statistical dissimilarities were detected among genetic clusters.

|  | ***Cluster 0*** | ***Cluster 1*** | ***Cluster 2*** | ***Cluster 3*** | ***Cluster 4*** |
| --- | --- | --- | --- | --- | --- |
| ***Cluster 0*** |  | **0.158** | **0.171** | **0.155** | **0.051** |
| ***Cluster 1*** | 1.247 |  | **0.223** | **0.217** | **0.056** |
| ***Cluster 2*** | 1.234 | 1.727 |  | **0.205** | **0.056** |
| ***Cluster 3*** | 1.105 | 1.663 | 1.554 |  | **0.044** |
| ***Cluster 4*** | 1.191 | 1.307 | 1.303 | 1.009 |  |

**SI Table 5** Number of reads obtained by *Illumina MiSeq* metabarcoding of *ITS* DNA samples, obtained from nine different date palm cultivars soils. Raw dataset was processed as described in Materials and Methods. Classified sequences correspond to those sequences classified as belonging to fungi before dataset subsampling.

| **Cultivar** | **Samples** | **Raw reads** | **Merged**  **reads** | **Processed**  **reads** | **Classified**  **sequences** |
| --- | --- | --- | --- | --- | --- |
| *Bejou* | BEJ1 | 321,864 | 132,808 | 122,960 | 111,214 |
|  | BEJ2 | 254,022 | 101,681 | 93,256 | 90,874 |
|  | BEJ3 | 315,166 | 122,821 | 112,566 | 111,214 |
|  | BEJ4 | 253,004 | 102,193 | 93,745 | 91,234 |
|  | **Total** | **1,144,056** | **459,503** | **422,527** | **404,536** |
| *Besser*  *Helou* | BEH1 | 271,008 | 107,250 | 98,186 | 96,036 |
|  | BEH2 | 131,506 | 58,809 | 54,420 | 52,893 |
|  | BEH3 | 153,974 | 67,106 | 61,532 | 58,253 |
|  | BEH4 | 268,964 | 107,301 | 96,604 | 88,188 |
|  | **Total** | **825,452** | **340466** | **310,742** | **295,370** |
| *Deglet*  *Nour* | DEN1 | 96,532 | 25,268 | 23,710 | 23,690 |
|  | DEN2 | 106,150 | 46,701 | 43,015 | 42,504 |
|  | DEN3 | 358,606 | 149,669 | 134,836 | 131,279 |
|  | DEN4 | 84,196 | 24,885 | 22,715 | 22,688 |
|  | **Total** | **645,484** | **246,523** | **224,276** | **220,170** |
| *Gondi* | GND1 | 164,550 | 73,945 | 67,650 | 66,946 |
|  | GND2 | 209,244 | 83,605 | 76,987 | 76,796 |
|  | GND3 | 162,560 | 67,745 | 61,291 | 61,126 |
|  | GND3 | 180,976 | 80,383 | 74,529 | 74,264 |
|  | **Total** | **717,330** | **305,678** | **280,457** | **279,132** |
| *Gosbi* | GSB1 | 130,177 | 52,071 | 47,652 | 47,067 |
|  | GSB2 | 253,685 | 101,474 | 92,951 | 87,726 |
|  | GSB3 | 243,414 | 49,961 | 46,441 | 45,961 |
|  | GSN4 | 108,542 | 41,998 | 37,892 | 37,379 |
|  | **Total** | **735,818** | **245,504** | **224,936** | **218,133** |
| *Kenta* | KT1 | 246,592 | 116,437 | 108,028 | 104,708 |
|  | KT2 | 225,580 | 107,221 | 97,974 | 92,547 |
|  | KT3 | 158,278 | 66,995 | 57,659 | 55,584 |
|  | KT4 | 214,962 | 88,519 | 82,011 | 80,483 |
|  | **Total** | **845,412** | **379,172** | **345,672** | **333,322** |
| *Lagou* | LAG1 | 445,208 | 205,525 | 177,292 | 158,157 |
|  | LAG2 | 242,988 | 84,974 | 76,594 | 66,665 |
|  | LAG3 | 303,080 | 121,607 | 110,227 | 108,307 |
|  | LAG4 | 263,008 | 105,682 | 97,929 | 97,240 |
|  | **Total** | **1,254,284** | **517,788** | **462,042** | **430,369** |
| *Tezerzayet*  *Kahla* | TZK1 | 232,206 | 110,262 | 100,774 | 100,008 |
|  | TZK2 | 301,058 | 143,083 | 129,593 | 125,512 |
|  | TZK3 | 234,662 | 111,116 | 102,042 | 99,162 |
|  | TZK4 | 174,078 | 83,049 | 75,791 | 73,331 |
|  | **Total** | **942,004** | **447,510** | **408,200** | **398,013** |
| *Tezerzayet*  *Safra* | TZS1 | 166,458 | 79,057 | 68,928 | 64,483 |
|  | TZS2 | 321,734 | 152,525 | 139,462 | 119,413 |
|  | TZS3 | 265,846 | 125,216 | 113,960 | 104,788 |
|  | TZS4 | 231,290 | 108,894 | 99,902 | 97,256 |
|  | **Total** | **985,328** | **465,692** | **422,252** | **385,940** |
| **Total** | | **8,095,168** | **3,407,836** | **3,101,104** | **2,964,985** |


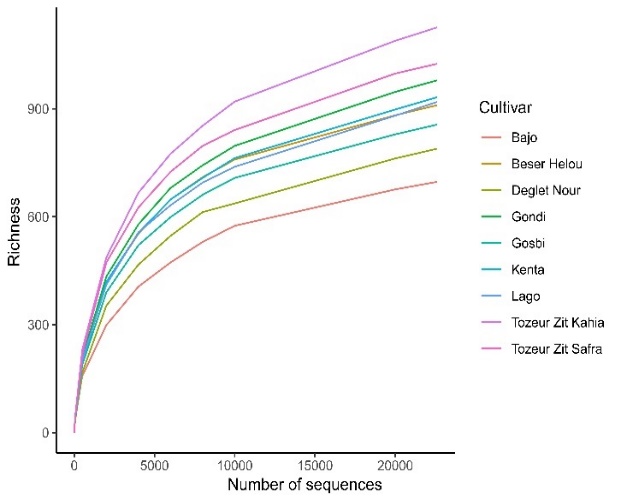

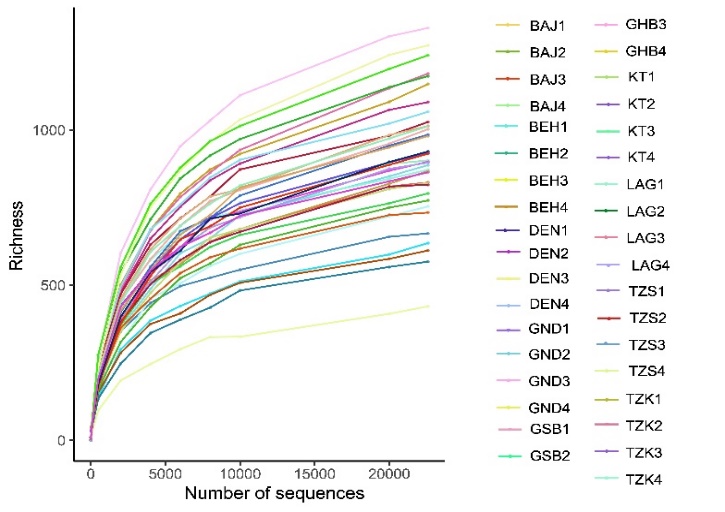

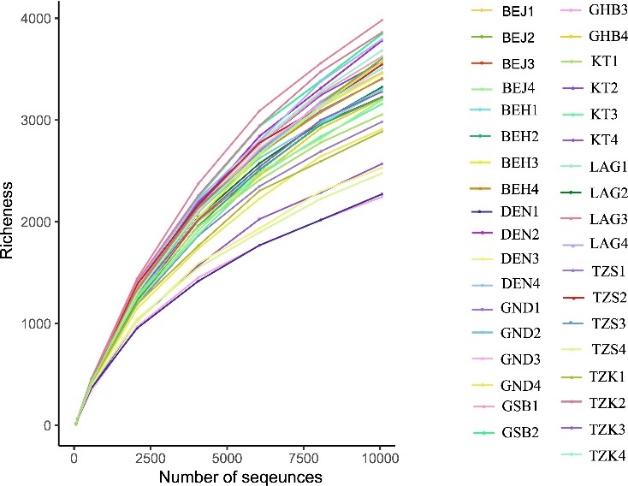

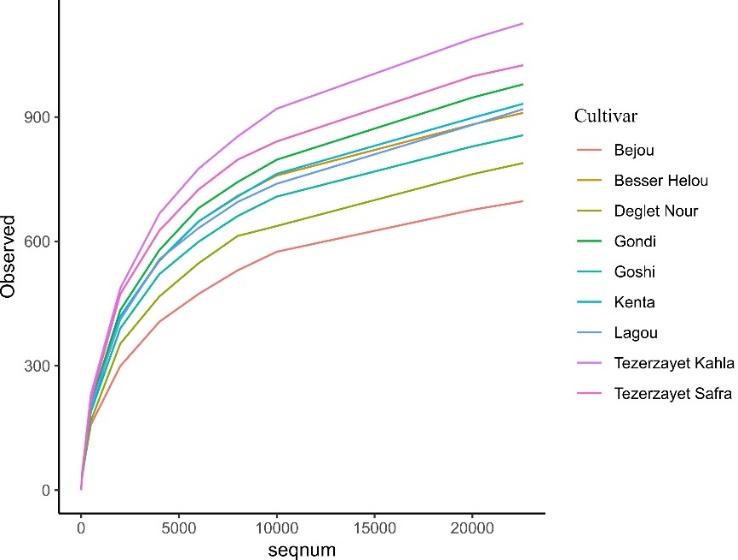


**a**

**b**

**SI Fig. 4** Rarefaction curves illustrating the diversity of bacterial communities, present in each date palm cultivar (a) and across distinct soil samples (b).

**SI Table 6** Richness (*S*) and alpha diversity indexes [Simpson’s index (1-*D*) and Shannon index (*H*’)] of fungal communities found in the soil of different date palm cultivars. Results are characterised by the mean value ± standard deviation (SD). Statistically differences at *p* < 0.05, among cultivars, are represented by different superscript letters. The values highlighted in bold represent the lowest values for richness and diversity, while those presented in bold and italics represent the highest values.

| **Cultivar** | **Richness (*S*)** | **Simpson’s index**  **(1-*D*)** | **Shannon’s index (*H’*)** |
| --- | --- | --- | --- |
| ***Bejou*** | **786 ± 147^c^** | 0.98 ± 8.60e^-03ab^ | 4.77 ± 0.30^bc^ |
| ***Besser Helou*** | 1,015 ± 134^abc^ | 0.97 ± 1.20e^-02ab^ | 5.09 ± 0.39^abc^ |
| ***Deglet Nour*** | 884 ± 285^bc^ | **0.96 ± 3.55e^-02b^** | **4.65 ± 0.90^c^** |
| ***Gondi*** | 1,107 ± 64^abc^ | 0.98 ± 6.70e^-03a^ | 5.32 ± 0.29^abc^ |
| ***Gosbi*** | 958 ± 222^abc^ | 0.98 ± 6.65e^-03ab^ | 5.15 ± 0.43^abc^ |
| ***Kenta*** | 1050 ± 152^abc^ | 0.98 ± 7.37e^-03ab^ | 5.23 ± 0.40^abc^ |
| ***Lagou*** | 1032 ± 176^abc^ | 0.99 ± 6.63e^-03a^ | 5.32 ± 0.39^abc^ |
| ***Tezer. Kahla*** | ***1,264 ± 167^a^*** | ***0.99 ± 3.30e^-03a^*** | ***5.65 ± 0.31^a^*** |
| ***Tezer. Safra*** | 1,145 ± 343^ab^ | 0.99 ± 7.04e^-03a^ | 5.48 ± 0.51^ab^ |

**SI Table 7** Permutational analysis of variance (PERMANOVA) of fungal communities found in date palm soils from each cultivar, using Bray-Curtis. Both *F* and *R^2^* (in bold) values are represented. Dissimilarities found among cultivars from ‘Bejou’, ‘Besser Helou’, ‘Kenta’, and ‘Lagou’ cluster are highlighted in light pink and those from ‘Gosbi’, ‘Gondi’, ‘Tezerzayet Safra’ and ‘Tezerzayet Kahla’ cluster in green. Statistical significance is denoted by an asterisk and was considered for *p* < 0.05.

|  | ***Bejou*** | ***Besser***  ***Helou*** | ***Deglet***  ***Nour*** | ***Gondi*** | ***Gosbi*** | ***Kenta*** | ***Lagou*** | ***Tezer.***  ***Kahla*** | ***Tezer.***  ***Safra*** |
| --- | --- | --- | --- | --- | --- | --- | --- | --- | --- |
| ***Bejou*** | - | **0.1736** | **0.2704** | **0.2410** | **0.2415** | **0.1523** | **0.1866** | **0.2073** | **0.1954** |
| ***Besser Helou*** | 1.2607 | - | **0.2317** | **0.2441** | **0.2390** | **0.1523** | **0.1807** | **0.2032** | **0.1612** |
| ***Deglet Nour*** | 2.2231* | 1.8091* | - | **0.3130** | **0.3107** | **0.2102** | **0.2724** | **0.2853** | **0.2106** |
| ***Gondi*** | 1.9047* | 1.9379* | 2.7331* | - | **0.2299** | **0.2294** | **0.1768** | **0.1838** | **0.2092** |
| ***Gosbi*** | 1.9100* | 1.8846* | 2.7039* | 1.7909* | - | **0.2558** | **0.2220** | **0.1944** | **0.1938** |
| ***Kenta*** | 1.0776 | 1.0782 | 1.5971 | 1.7858 | 2.0942* | - | **0.1759** | **0.1842** | **0.1471** |
| ***Lagou*** | 1.3776* | 1.3234 | 2.2462* | 1.2893 | 1.7121 | 1.2806 | - | **0.1696** | **0.1869** |
| ***Tezer. Kahla*** | 1.5688* | 1.5300* | 2.3961* | 1.3513 | 1.4484* | 1.3550 | 1.2261 | - | **0.1369** |
| ***Tezer. Safra*** | 1.4571* | 1.1527 | 1.6001 | 1.5871* | 1.4424 | 1.0345 | 1.3793* | 0.9522 | - |

**SI Table 8** Permutational analysis of variance (PERMANOVA) of fungal communities found in the different genetic clusters formed by the different date palm cultivars, using Bray-Curtis dissimilarities. Both *F* and *R*^2^ (in bold) values are represented. Cluster 2, composed by ‘Deglet Nour’, appears to be significantly distant from the other genetic clusters. Statistical significance is denoted by an asterisk and was considered for *p* < 0.05.

|  | ***Cluster 0*** | ***Cluster 1*** | ***Cluster 2*** | ***Cluster 3*** | ***Cluster 4*** |
| --- | --- | --- | --- | --- | --- |
| ***Cluster 0*** |  | **0.137** | **0.210** | **0.161** | **0.058** |
| ***Cluster 1*** | 0.952 |  | **0.285** | **0.203** | **0.0528** |
| ***Cluster 2*** | 1.600 | 2.396* |  | **0.231** | **0.090** |
| ***Cluster 3*** | 1.152 | 1.530 | 1.800* |  | **0.062** |
| ***Cluster 4*** | 1.365 | 1.226 | 2.174** | 1.460 |  |


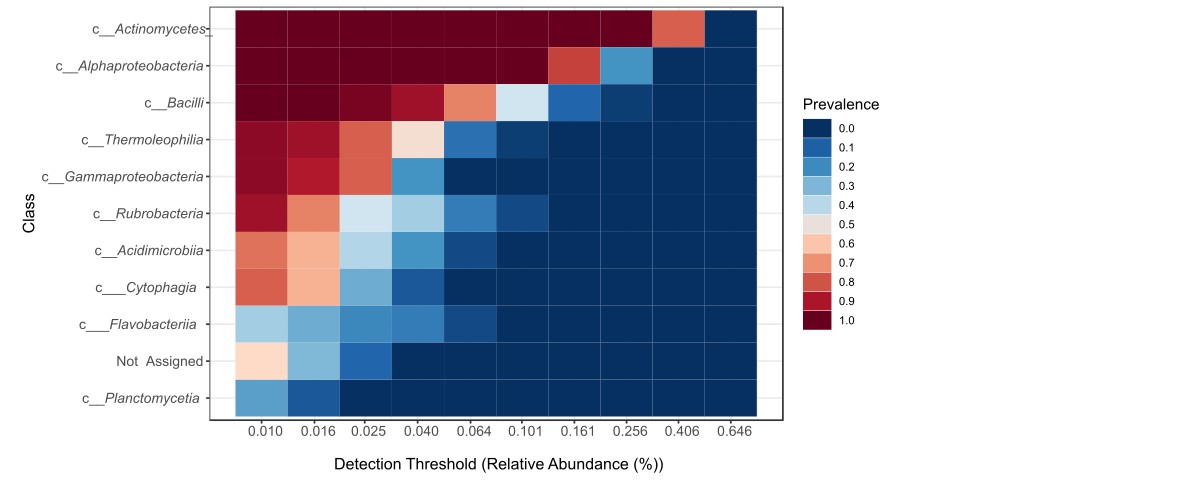


**SI Fig. 5** Date palm core microbiome based on relative abundance and sample prevalence (20%) of bacterial classes. Colour shading indicates the prevalence of each bacterial class among samples for each abundance threshold.

**SI Table 9** Relative abundance of the bacterial classes that were significantly different among distinct cultivar communities. Different letters mean statistical differences, determined by ANOVA (*p* ≤ 0.001)

|  | ***Bejou*** | ***Besser Helou*** | ***Deglet Nour*** | ***Gondi*** | ***Gosbi*** | ***Kenta*** | ***Lagou*** | ***Tezer. Kahla*** | ***Tezer. Safra*** |
| --- | --- | --- | --- | --- | --- | --- | --- | --- | --- |
| ***Abditibacteria*** | 0.005^ab^ | 0.003^b^ | 0.002^b^ | 0.003^b^ | 0.003^b^ | 0.004^b^ | 0.003^b^ | 0.007^a^ | 0.003^b^ |
| ***Acidobacteria*** | 0.004^ab^ | 0.003^ab^ | 0.002^b^ | 0.003^ab^ | 0.004^ab^ | 0.003^ab^ | 0.004^ab^ | 0.006^a^ | 0.004^ab^ |
| ***Alphaproteobacteria*** | 0.227^ab^ | 0.225^ab^ | 0.205^b^ | 0.215^ab^ | 0.223^ab^ | 0.225^ab^ | 0.228^ab^ | 0.273^a^ | 0.209^ab^ |
| ***Anaerolineae*** | 0.002^ab^ | 0.003^a^ | 0.001^bc^ | 0.002^abc^ | 0.002^abc^ | 0.001^bc^ | 0.002^abc^ | 0.0006^c^ | 0.0009^c^ |
| ***Bdellovibrionia*** | 0.001^b^ | 0.001^b^ | 0.003^ab^ | 0.003^ab^ | 0.002^ab^ | 0.003^ab^ | 0.003^a^ | 0.002^ab^ | 0.002^ab^ |
| ***Blastocatellia*** | 0.001^b^ | 0.001^b^ | 0.002^b^ | 0.005^a^ | 0.003^ab^ | 0.0001^b^ | 0.0006^b^ | 0.0008^b^ | 0.0002^b^ |
| ***Chitinophagia*** | 0.003^ab^ | 0.003^ab^ | 0.003^b^ | 0.004^ab^ | 0.004^a^ | 0.003^ab^ | 0.004^ab^ | 0.002^ab^ | 0.001^b^ |
| ***Chlamydiae*** | 0.001^ab^ | 0.001^ab^ | 0.001^b^ | 0.001^b^ | 0.001^ab^ | 0.004^ab^ | 0.003^ab^ | 0.003^ab^ | 0.005^a^ |
| ***Chloroflexia*** | 0.015^ab^ | 0.012^ab^ | 0.008^b^ | 0.011^ab^ | 0.009^ab^ | 0.013^ab^ | 0.014^ab^ | 0.012^ab^ | 0.016^a^ |
| ***Cytophagia*** | 0.021^b^ | 0.021^b^ | 0.030^b^ | 0.029^b^ | 0.027^b^ | 0.032^b^ | 0.026^b^ | 0.051^a^ | 0.029^b^ |
| ***Dehalococcoidia*** | 0.002^a^ | 0.002^a^ | 0.0004^b^ | 0.001^ab^ | 0.0008^ab^ | 0.003^a^ | 0.002^ab^ | 0.0009^ab^ | 0.002^ab^ |
| ***Entotheonellia*** | 0.003^ab^ | 0.005^a^ | 0.001^b^ | 0.003^ab^ | 0.002^b^ | 0.001^b^ | 0.002^b^ | 0.002^b^ | 0.002^b^ |
| ***Gemmatimonadetes*** | 0.006^abc^ | 0.008^ab^ | 0.002^c^ | 0.003^bc^ | 0.006^abc^ | 0.007^abc^ | 0.006^abc^ | 0.009^a^ | 0.007^ab^ |
| ***Longimicrobia*** | 0.016^bc^ | 0.015^bcd^ | 0.011^cd^ | 0.008^d^ | 0.007^d^ | 0.027^a^ | 0.015^bcd^ | 0.019^bc^ | 0.022^ab^ |
| ***Phycisphaerae*** | 0.003^ab^ | 0.002^ab^ | 0.002^b^ | 0.003^ab^ | 0.004^a^ | 0.001^b^ | 0.002^ab^ | 0.002^b^ | 0.001^b^ |
| ***Planctomycetes*** | 0.022^ab^ | 0.025^a^ | 0.017^ab^ | 0.026^a^ | 0.028^a^ | 0.013^ab^ | 0.021^ab^ | 0.006^b^ | 0.007^b^ |
| ***Rhodothermia*** | 0.005^b^ | 0.005^ab^ | 0.008^ab^ | 0.004^b^ | 0.004^b^ | 0.007^ab^ | 0.006^ab^ | 0.005^b^ | 0.012^a^ |
| ***Verrucomicrobiae*** | 0.007^ab^ | 0.008^ab^ | 0.004^b^ | 0.010^a^ | 0.008^ab^ | 0.004^b^ | 0.006^ab^ | 0.006^ab^ | 0.005^ab^ |
| ***Vicinamibacteria*** | 0.002^abc^ | 0.003^a^ | 0.001^bcd^ | 0.002^abcd^ | 0.003^ab^ | 0.0006^cd^ | 0.002^abcd^ | 0.0005^d^ | 0.0004^d^ |

**SI Table 10** The relative abundance of the 6 most abundant bacterial genera is represented. Relative abundance of the different top-6 most abundant bacterial genera among distinct cultivar communities. Different letters mean statistical differences, determined by ANOVA (*p* ≤ 0.001).

| Genus | | ***Bejou*** | ***Besser Helou*** | ***Deglet Nour*** | ***Gondi*** | ***Gosbi*** | ***Kenta*** | ***Lagou*** | ***Tezer. Kahla*** | ***Tezer. Safra*** |
| --- | --- | --- | --- | --- | --- | --- | --- | --- | --- | --- |
|  | ***Bacillus*** | 0.06^a^ | 0.03^a^ | 0.04^a^ | 0.05^a^ | 0.05^a^ | 0.02^a^ | 0.05^a^ | 0.02^a^ | 0.02^a^ |
|  | ***Blastococcus*** | 0.04^a^ | 0.05^a^ | 0.04^a^ | 0.03^a^ | 0.04^a^ | 0.04^a^ | 0.05^a^ | 0.03^a^ | 0.03^a^ |
|  | ***Kocuria*** | 0.03^b^ | 0.02^b^ | 0.09^a^ | 0.03^b^ | 0.02^b^ | 0.06^ab^ | 0.03^b^ | 0.02^b^ | 0.02^b^ |
|  | ***Geodermatophilus*** | 0.04^a^ | 0.04^ab^ | 0.02^b^ | 0.03^ab^ | 0.02^b^ | 0.03^ab^ | 0.04^ab^ | 0.02^b^ | 0.02^b^ |
|  | ***Microvirga*** | 0.04^ab^ | 0.03^ab^ | 0.02^b^ | 0.02^b^ | 0.03^ab^ | 0.02^b^ | 0.02^b^ | 0.05^a^ | 0.02^b^ |
|  | ***Nocardioides*** | 0.02^ab^ | 0.03^ab^ | 0.02^ab^ | 0.04^ab^ | 0.04^a^ | 0.02^b^ | 0.03^ab^ | 0.03^ab^ | 0.02^b^ |


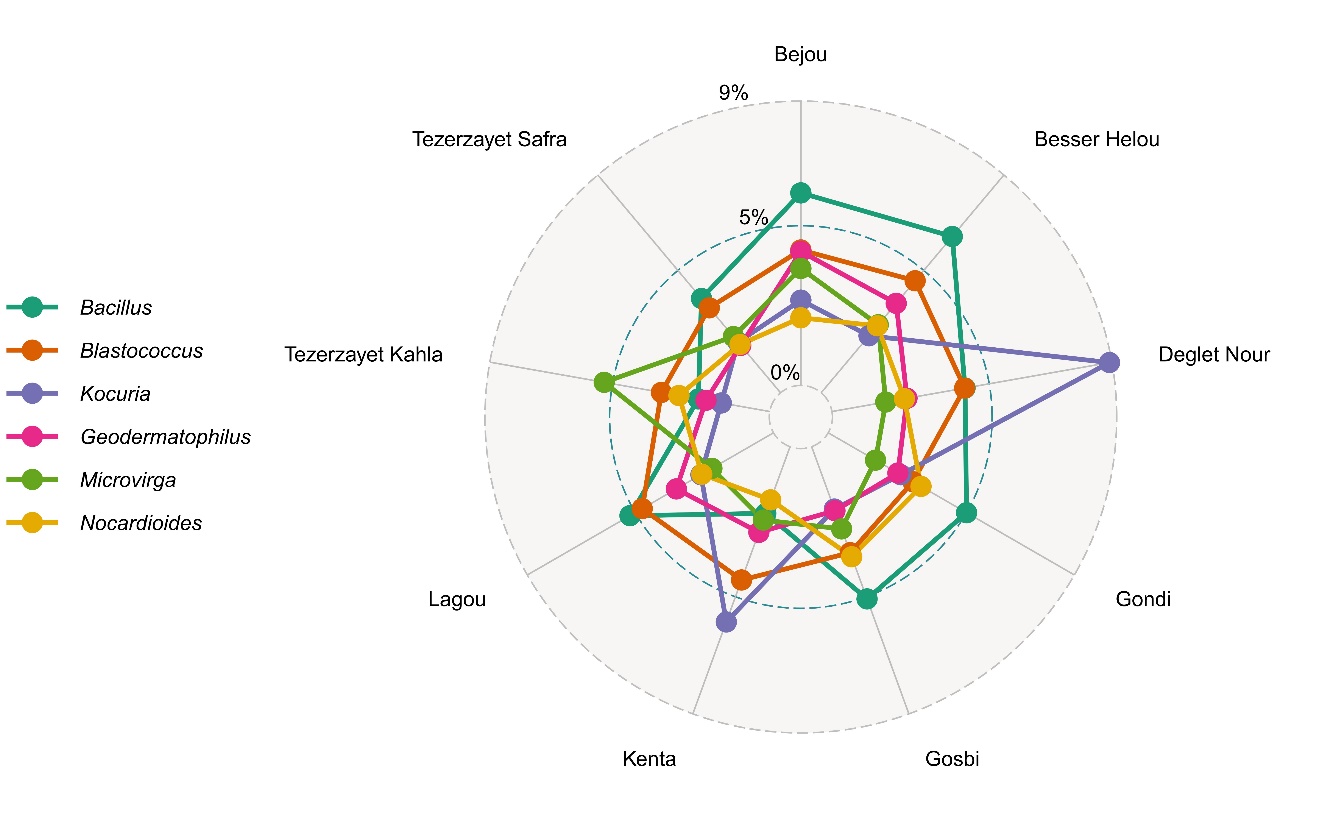


**SI Fig. 6** Relative abundance of the top-6 bacterial genera found in different date palm cultivars.


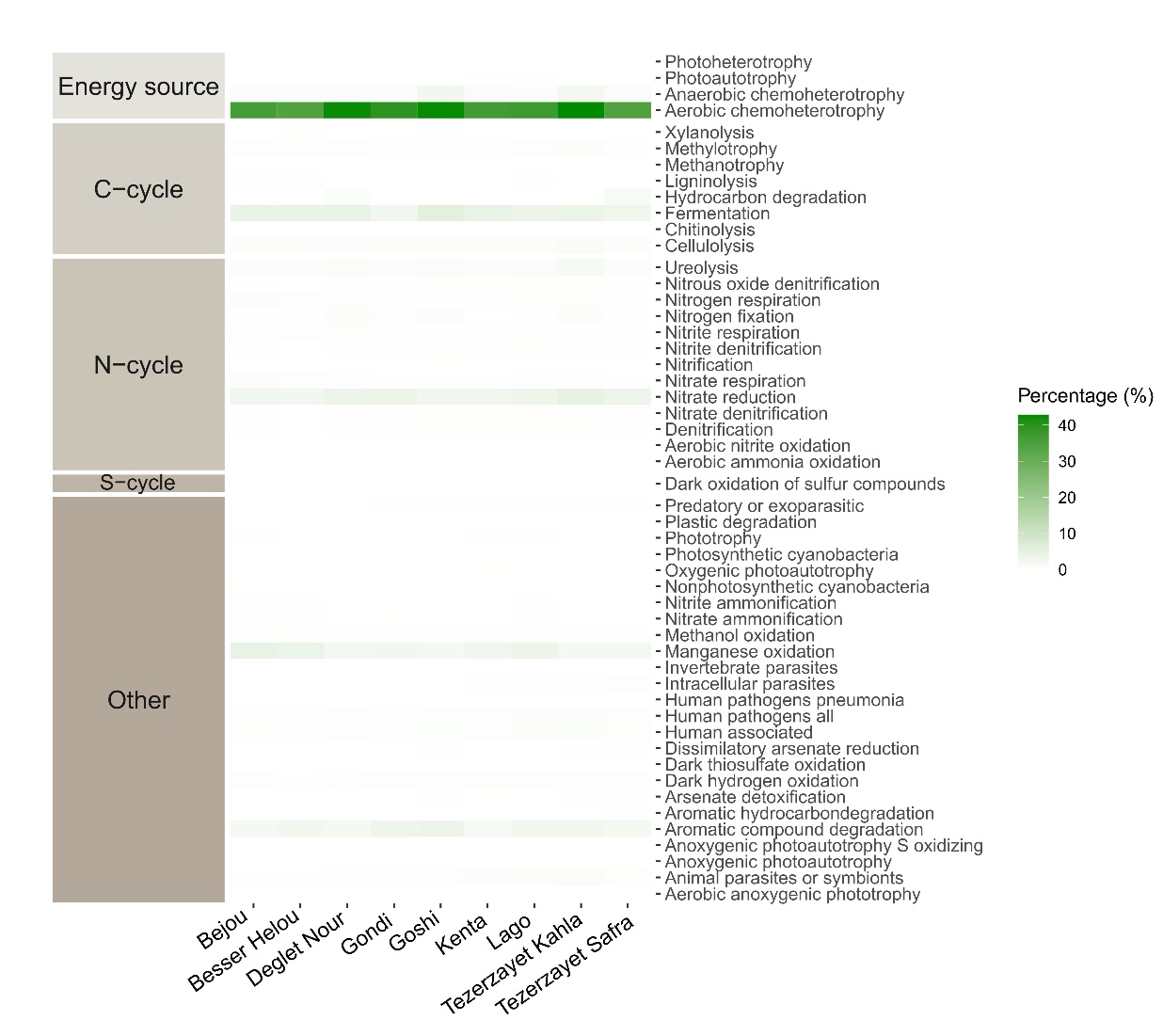


**SI Fig. 7** Date palm functional groups for soil bacterial community

**SI Table 11** Relative abundance of the different classes of fungi among different cultivar communities. Different letters mean statistical differences, determined by ANOVA (*p* ≤ 0.05).

|  | ***Bejou*** | ***Besser Helou*** | ***Deglet Nour*** | ***Gondi*** | ***Gosbi*** | ***Kenta*** | ***Lagou*** | ***Tezer. Kahla*** | ***Tezer. Safra*** |
| --- | --- | --- | --- | --- | --- | --- | --- | --- | --- |
| **Agaricomycetes** | 0.015^b^ | 0.034^ab^ | 0.052^ab^ | 0.030^ab^ | 0.059^ab^ | 0.0284^ab^ | 0.080^ab^ | 0.101^a^ | 0.098^a^ |
| **Arthoniomycetes** | 0^b^ | 0.0004^b^ | 0.003^b^ | 0.003^b^ | 0.0003^b^ | 0.003^b^ | 8.152e-05^b^ | 0.004^b^ | 0.023^a^ |
| **Chytridiomycetes** | 0.0008^ab^ | 0.0004^b^ | 0.0003^b^ | 7.713e-05^b^ | 0.004^a^ | 0.0005^b^ | 0.0008^ab^ | 0.002^ab^ | 0.003^ab^ |
| **Cystobasidiomycetes** | 0.0004^c^ | 0.002^bc^ | 0.002^bc^ | 0.002^bc^ | 0.003^bc^ | 0.003^bc^ | 0.001^c^ | 0.009^ab^ | 0.010^a^ |
| **Dothideomycetes** | 0.222^b^ | 0.256^b^ | 0.546^a^ | 0.279^b^ | 0.272^b^ | 0.367^b^ | 0.279^b^ | 0.279^b^ | 0.36^b^ |
| **Eurotiomycetes** | 0.210^a^ | 0.057^b^ | 0.148^ab^ | 0.133^ab^ | 0.089^b^ | 0.112^ab^ | 0.120^ab^ | 0.121^ab^ | 0.081^b^ |
| **Orbiliomycetes** | 2.203e^-05b^ | 1.102e^-05b^ | 0.001^b^ | 0.017^a^ | 0.002^ab^ | 0.0002^b^ | 0.0004^b^ | 0.0009^b^ | 0.008^ab^ |
| **Pezizomycetes** | 0.148^ab^ | 0.204^a^ | 0.024^b^ | 0.040^b^ | 0.041^b^ | 0.109^ab^ | 0.064^b^ | 0.064^b^ | 0.063^b^ |
| **Sordariomycetes** | 0.170^ab^ | 0.298^a^ | 0.080^b^ | 0.325^a^ | 0.197^ab^ | 0.176^ab^ | 0.316^a^ | 0.214^ab^ | 0.165^ab^ |
| **Tremellomycetes** | 0.122^a^ | 0.027^b^ | 0.023^b^ | 0.070^ab^ | 0.121^a^ | 0.048^ab^ | 0.044^ab^ | 0.042^ab^ | 0.073^ab^ |
| **Ustilaginomycetes** | 0.0002^b^ | 0.0001^b^ | 3.306e^-05b^ | 0.0003^b^ | 0.0006^b^ | 0.021^a^ | 0.003^b^ | 0.009^ab^ | 0.003^b^ |
| **Wallemiomycetes** | 0.001^b^ | 3.306e^-05b^ | 0.0002^b^ | 0.016^b^ | 0.0002^b^ | 2.204e-05^a^ | 6.611e^-05b^ | 0.002^b^ | 0.0002^b^ |


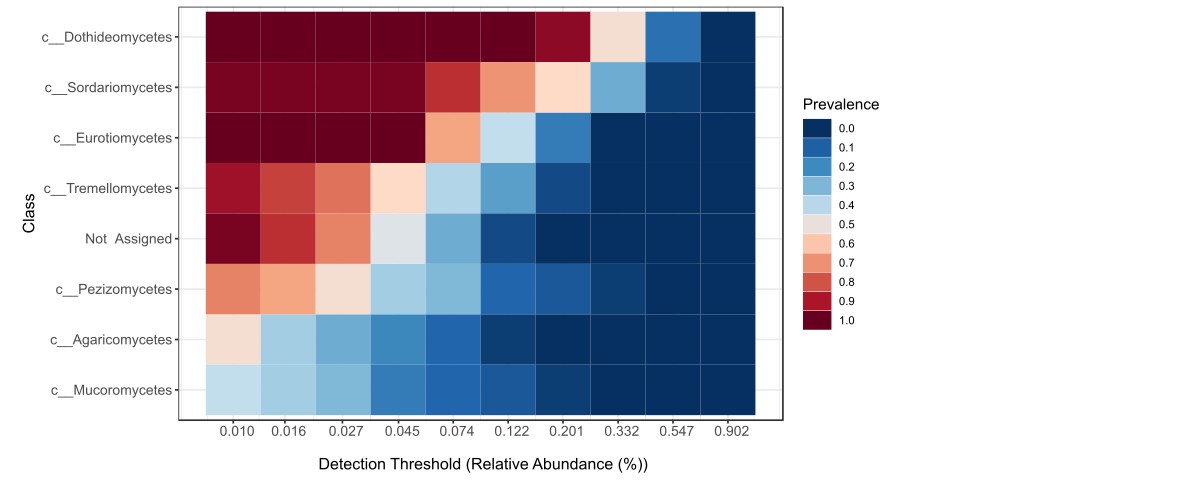


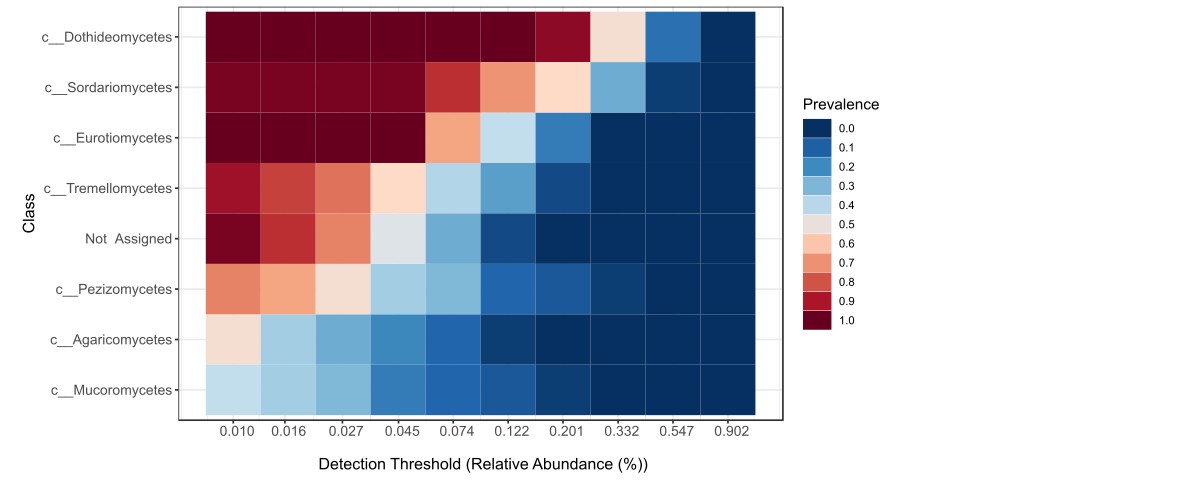


**SI Fig. 8** Date palm core microbiome analysis based on relative abundance and sample prevalence (20%) of fungal classes. Colour shading indicates the prevalence of each fungal class among samples for each abundance threshold

**B**


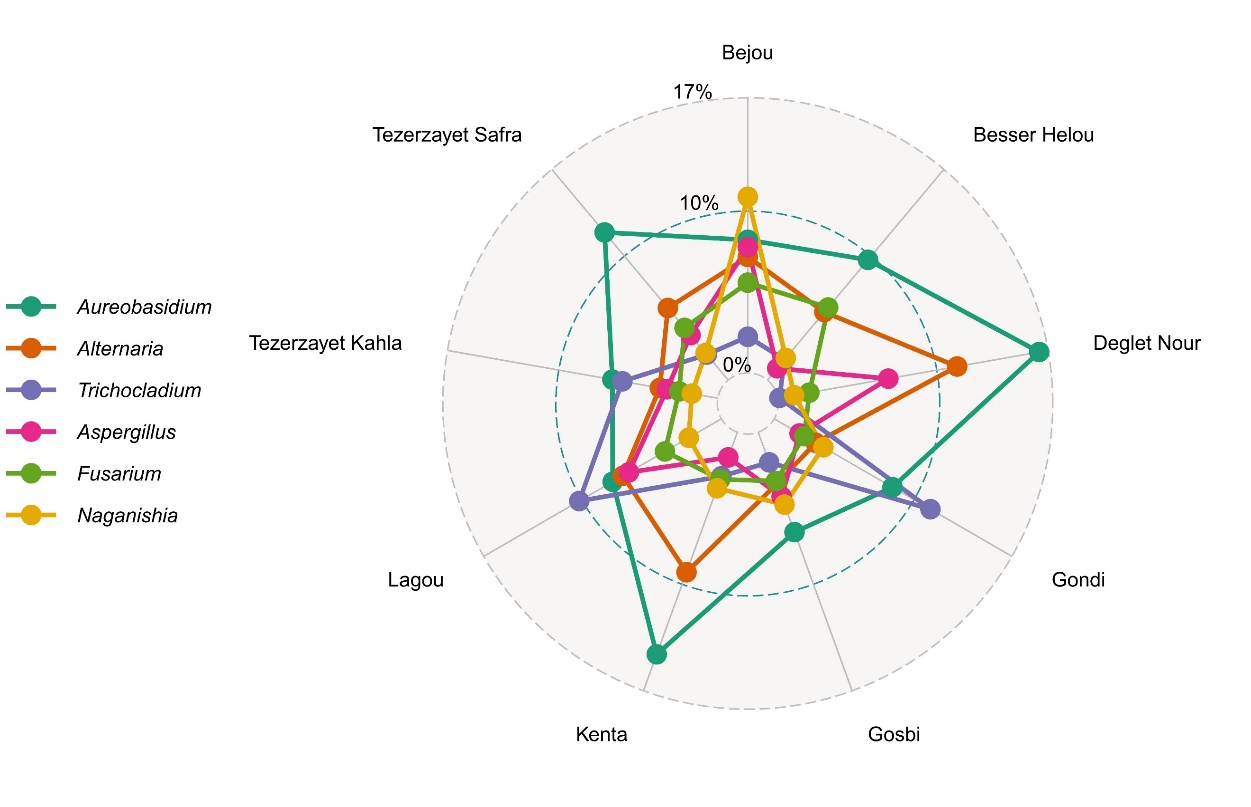


**SI Fig. 9** Relative abundance of the top-6 fungal genera found in different date palm cultivars.

| Genus | | ***Bejou*** | ***Besser Helou*** | ***Deglet Nour*** | ***Gondi*** | ***Gosbi*** | ***Kenta*** | ***Lagou*** | ***Tezer. Kahla*** | ***Tezer. Safra*** |
| --- | --- | --- | --- | --- | --- | --- | --- | --- | --- | --- |
|  | ***Aureobasidium*** | 0.08^a^ | 0.10^a^ | 0.16^a^ | 0.08^a^ | 0.07^a^ | 0.15^a^ | 0.08^a^ | 0.07^a^ | 0.12^a^ |
|  | ***Alternaria*** | 0.07^abc^ | 0.05^abc^ | 0.11^a^ | 0.03^c^ | 0.03^bc^ | 0.09^ab^ | 0.07^abc^ | 0.04^bc^ | 0.06^abc^ |
|  | ***Trichocladium*** | 0.02^c^ | 0.02^c^ | 0.0009^c^ | 0.11^a^ | 0.02^c^ | 0.03^bc^ | 0.10^ab^ | 0.06^abc^ | 0.02^c^ |
|  | ***Aspergillus*** | 0.03^a^ | 0.004^c^ | 0.02^ab^ | 0.01^c^ | 0.04^abc^ | 0.02^c^ | 0.07^ab^ | 0.03^bc^ | 0.04^bc^ |
|  | ***Fusarium*** | 0.06^a^ | 0.06^a^ | 0.02^a^ | 0.02^a^ | 0.03^a^ | 0.03^a^ | 0.04^a^ | 0.02^a^ | 0.04^a^ |
|  | ***Naganishia*** | 0.10^a^ | 0.02^b^ | 0.01^b^ | 0.04^b^ | 0.05^b^ | 0.04^b^ | 0.02^b^ | 0.02^b^ | 0.02^b^ |

**SI Table 12** The relative abundance of the 6 most abundant fungal genera is represented. Relative abundance of the different top-6 most abundant fungal genera among distinct cultivar communities. Different letters mean statistical differences, determined by ANOVA (*p* ≤ 0.001).


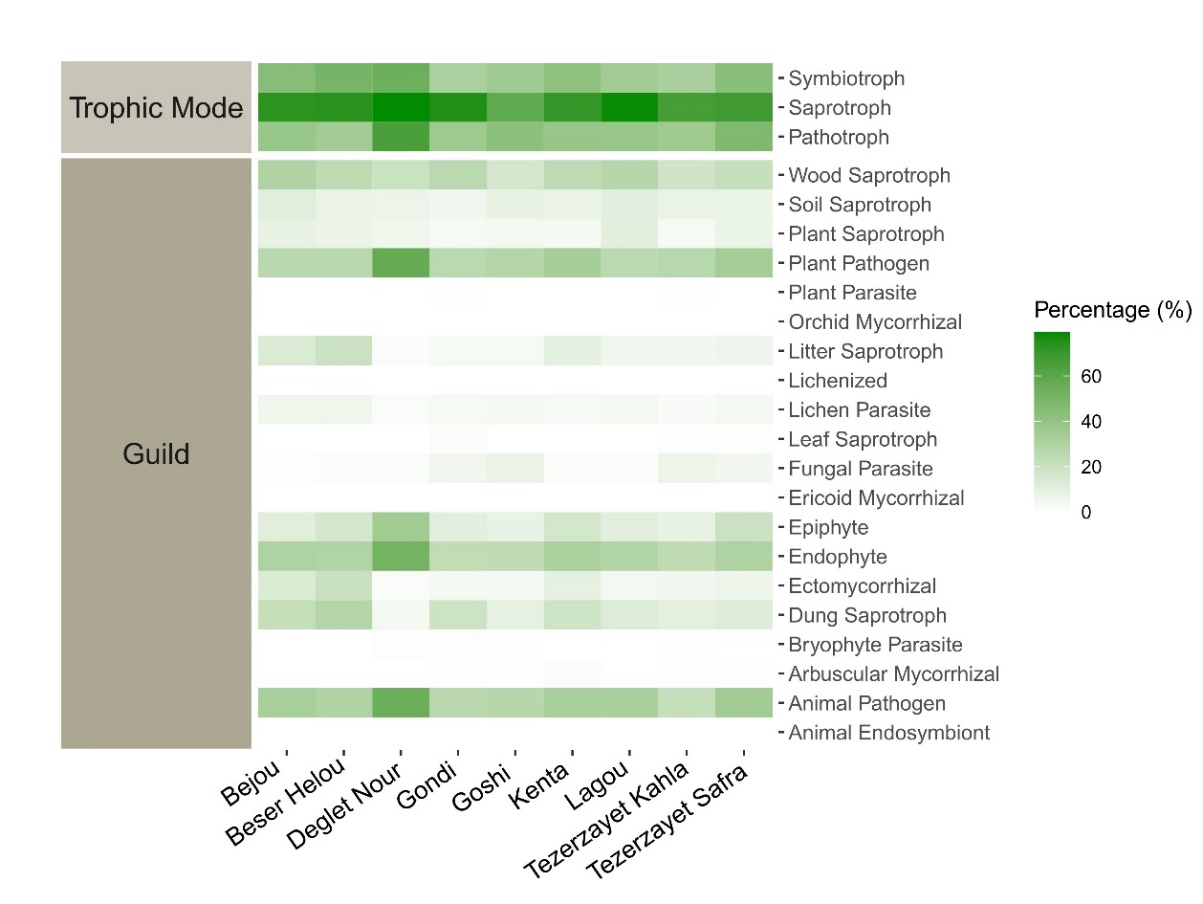


**SI Fig. 10** Date palm functional groups for soil fungal communities.

**a**

**b**


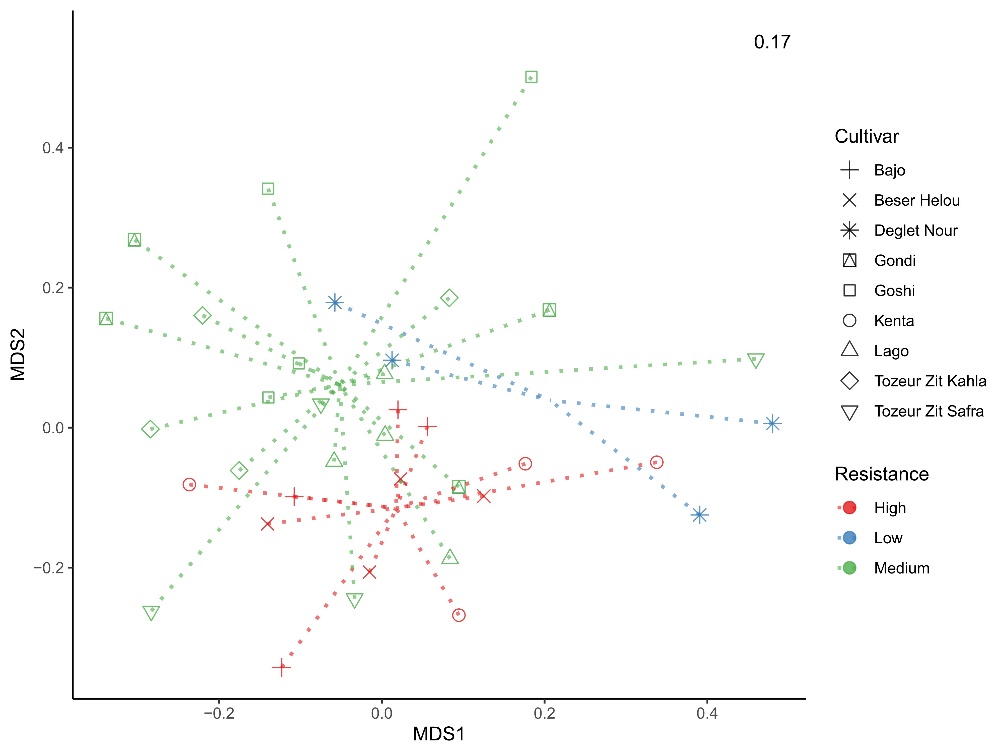

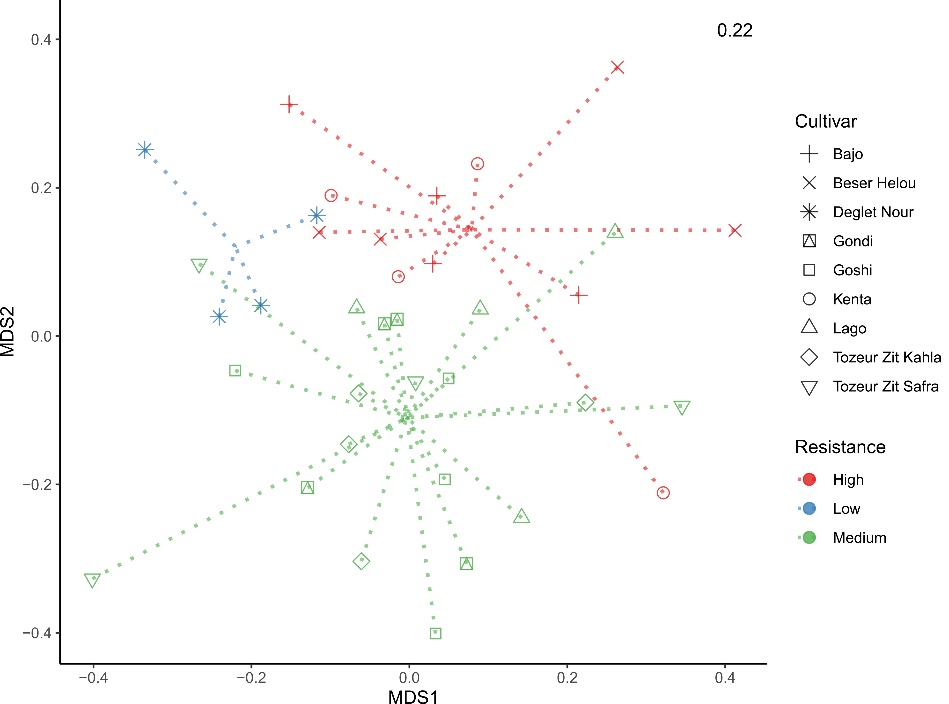


**SI Fig. 11** Non-metric multidimensional scale (NMDS) plots for bacterial (a) and fungal (b) communities found in the soils from different date palm cultivars. Clustering was performed using the Bray-Curtis dissimilarity. Each data point corresponds to a sample from a specific date palm cultivar, distinguished by shape, the different colours represent the different levels of resistance to stress as indicated in the legend.
